# Gut microbiota in regulatory T cell generation and function: mechanisms and health implications

**DOI:** 10.1080/19490976.2025.2516702

**Published:** 2025-06-15

**Authors:** Amit Sharma, Garima Sharma, Sin-Hyeog Im

**Affiliations:** a Department of Life Sciences, Pohang University of Science and Technology (POSTECH), Pohang, Republic of Korea; b Innovation Research Center for Bio-Future Technology (B-IRC), Pohang University of Science and Technology (POSTECH), Pohang, Republic of Korea; c ImmunoPharm Group, ImmmunoBiome Inc, Pohang, Republic of Korea; d Institute for Convergence Research and Education in Advanced Technology, Yonsei University, Seoul, Republic of Korea

**Keywords:** Microbiome, regulatory T cells, live biotherapeutic products, microbial ligands, microbial metabolites, dysbiosis

## Abstract

The establishment and maintenance of immune homeostasis rely on a dynamic, bidirectional exchange of information between commensal microorganisms and the host immune system. At the center of this process are CD4^+^Foxp3^+^ regulatory T cells (Tregs), which have emerged as pivotal mediators to ensure immunological equilibrium. This review explores the sophisticated mechanisms by which the gut microbiota modulates the differentiation, expansion, and functional specialization of Tregs, orchestrating intestinal immune tolerance to support host-microbiota mutualism. We discuss the role of microbial-derived structural components and metabolites in shaping the immunoregulatory fitness of Tregs. Additionally, we explore the impact of gut microbial dysbiosis, where disrupted microbial-immune crosstalk compromises immune tolerance, contributing to the development of inflammatory and autoimmune disorders. Finally, we highlight the potential of microbiota-based strategies to recalibrate intestinal immunity and restore immune tolerance.

## Introduction

The human body represents a complex ecosystem, intricately intertwined with trillions of microorganisms that collectively constitute the gut microbiome – a dynamic consortium encompassing bacteria, viruses, fungi, and other microbial entities. This microbial community is dominated by two bacterial phyla, *Bacteroidetes* and *Firmicutes*, which constitute approximately 90% of the total gut microbial biomass, with smaller contributions from *Proteobacteria*, *Actinobacteria*, and *Verrucomicrobia*.^1–3^ Commensal fungi, such as *Candida* and *Saccharomyces* species, and *bacteriophages* further contribute to this ecosystem, while gut-resident bacteria reciprocally suppress pathogenic invaders. While the human genome encodes approximately 20,000 genes, the hologenome, which integrates the host genome with the collective genetic material of its resident microbiota, comprises over 33 million genes.^4^ This vast genetic reservoir enables the gut microbiota to establish a mutualistic relationship with the host, performing critical functions such as fermenting dietary fibers to produce short-chain fatty acids (SCFAs),^5–7^ synthesizing vitamins (e.g., vitamin B12 and K),^8,9^ metabolizing xenobiotics,^10^ and competitively excluding pathogens,^11^ while the human gut provides protection, nutrients, and favorable growth conditions for these microbes.

Additionally, the gut microbiome has emerged as a central regulator of host immunity, profoundly influencing immune development, tolerance, and homeostasis influencing both innate and adaptive immune responses.^5,12^ Dysbiosis – alterations in microbial composition or function – has been linked to numerous diseases, including inflammatory bowel disease (IBD), autoimmune disorders, and cancer.^5,12–16^

For this mutualistic relationship to thrive, the host needs to recognize the microbiome as part of itself, a process facilitated by co-evolved mechanisms that ensure immune tolerance. The gut immune system comprises a highly specialized and compartmentalized network of innate and adaptive immune components that work synergistically to maintain mucosal homeostasis and defend against pathogens. Key innate immune cells involved in mucosal defense include intestinal epithelial cells (IECs) and innate lymphoid cells (ILCs). IECs act as a physical barrier and also produce antimicrobial peptides and cytokines. Among the ILCs, group 3 ILCs (ILC3s) are especially important for maintaining mucosal immune homeostasis and promoting tolerance to commensal microbes.^17,18^ Dendritic cells (DCs) and macrophages continuously sample luminal antigens and help orchestrate immune responses, often promoting regulatory over inflammatory pathways.^19,20^ Among adaptive components, IgA-producing plasma cells are essential for neutralizing pathogens and shaping microbial composition without inducing inflammation.^21^ Importantly, the gut harbors a substantial population of type 1 regulatory (Tr1) T cells that secrete high levels of IL-10 and do not express Foxp3 constitutively.^22^ Additionally, regulatory B cells that produce IL-10 and TGF-β to suppress inflammatory responses.^23^ Foxp3^+^CD4^+^ regulatory T cells (Tregs) and Th17 cells, whose balance is crucial for immune tolerance and pathogen defense, respectively.^12^

Tregs, a specialized subset of CD4^+^ T cells characterized by the expression of the transcription factor Foxp3, play a crucial role in maintaining immune homeostasis and preventing excessive inflammatory responses.^24,25^ Tregs are indispensable for establishing dominant immune tolerance and maintaining immune homeostasis. Tregs exert their suppressive functions through multiple mechanisms, including the production of anti-inflammatory cytokines (IL-10, TGF-β, IL-35), metabolic disruption of effector T cells, cytolysis, and modulation of dendritic cell function.^24,25^ They are broadly classified into two categories: thymic Tregs (tTregs), which develop in the thymus and prevent autoimmunity, and peripheral Tregs (pTregs), which differentiate in peripheral tissues and mediate tolerance to innocuous antigens, including dietary components and commensal microbes.^15,26^ Within the intestinal mucosa, a significant population of Tregs co-expresses Foxp3 and RORγt, the latter being a transcription factor typically associated with Th17 cells.^15^ These RORγt+ Tregs are predominantly of peripheral origin and play a crucial role in maintaining tolerance to the gut microbiota.^26^ Their development and maintenance are heavily influenced by microbial signals, highlighting the intimate relationship between the gut microbiota and the regulatory arm of the immune system.

The gut microbiome has evolved sophisticated mechanisms to influence the differentiation, expansion, and functional fitness of Tregs. In turn, Tregs suppress excessive immune responses, thereby preserving the diversity and eubiosis of the commensal microbiota. This reciprocal interaction underscores the critical importance of the microbiome-Treg axis in immune regulation. Recent advances have revealed that microbial structural components, such as polysaccharide A (PSA), cell surface β-glucan/galactan polysaccharides (CSGG), and mannan/β-1,6-glucan-containing polysaccharides (MGCP), directly modulate Treg differentiation and function.^27–29^ Furthermore, microbial metabolites – including short-chain fatty acids (SCFAs), tryptophan derivatives, and BA – play pivotal roles in shaping Treg biology through epigenetic modifications, metabolic reprogramming, and receptor-mediated signaling pathways.^30–32^ However, dysregulation of the microbiome-Treg axis can lead to immune dysfunction, contributing to the pathogenesis of inflammatory and autoimmune diseases. For example, in inflammatory bowel disease (IBD), dysbiosis and reduced production of SCFAs and secondary BA impair Treg function, resulting in chronic inflammation.^13,33^

In this review, we explore microbial factors and mechanisms that support Treg function in maintaining immune homeostasis. We also examine the therapeutic potential of targeting the microbiome-Treg axis in the context of inflammatory and autoimmune diseases. By integrating recent advances, we highlight the pivotal role of microbially derived signals in immune regulation and their implications for disease prevention and treatment.

## Microbial modulation of tregs

The incorporation of gut microbes into the host’s immunological self requires the establishment of active immune tolerance to prevent inappropriate immune activation while preserving the ability to respond to harmful pathogens. Indeed, Germ-free (GF) mice, which lack a gut microbiome, exhibit an underdeveloped immune system, highlighting the critical role of microbial colonization in immune maturation.^5^ Furthermore, the depletion of microbiota with oral antibiotics has been shown to exacerbate intestinal inflammation, underscoring the importance of the gut microbiome in maintaining peripheral tolerance.^34^ Tregs have been extensively studied toward establishment of central and peripheral immune tolerance, since their discovery and, over the last two decades, have emerged as central regulators in establishing and maintaining dominant immune tolerance.^35^ As described above, two major subtypes of Tregs – tTregs and pTregs were initially thought to have distinct roles, recent studies suggest that both subsets can be induced in response to microbial antigens, challenging their traditional classifications.^36,37^

The gut microbiota plays a critical role in shaping Treg populations, both in the thymus and the periphery. Microbial-derived signals, including polysaccharides, metabolites, and structural components, directly influence Treg differentiation, expansion, and function.^5,31^ These interactions highlight the intricate crosstalk between the microbiota and Tregs, which is essential for maintaining immune homeostasis and preventing inflammatory diseases.

While the gut microbiota is a critical regulator of Treg-mediated immune tolerance, not all microbial species or their metabolites universally promote anti-inflammatory responses. Certain gut bacteria, such as Segmented Filamentous Bacteria (SFB), are known to drive pro-inflammatory Th17 cell responses, which can exacerbate inflammation in susceptible hosts.^34,38^ For instance, SFB colonization in mice has been shown to promote Th17 cell differentiation in the gut, contributing to autoimmune conditions such as experimental autoimmune encephalomyelitis (EAE).^39^ Similarly, *Prevotella copri* has been associated with enhanced susceptibility to colitis and arthritis through activation of pro-inflammatory pathways.^40,41^ Pathobionts such as *Enterococcus faecalis* and adherent-invasive *Escherichia coli* (AIEC) exacerbate inflammatory bowel disease (IBD) by activating NF-κB and NLRP3 inflammasome pathways, thereby suppressing Treg activity.^42–44^ Even commensals like Helicobacter hepaticus can adopt pathogenic roles in genetically susceptible hosts, triggering colitis through IL-23-driven Th17 responses.^45^ These examples underscore the context-dependent nature of microbial-immune interactions, where the same microbiota can either promote tolerance or inflammation depending on host genetics, microbial strain specificity, and environmental triggers. In this section, we explore the mechanisms by which the relevant gut microbiota modulates Treg biology, focusing on the generation and function of both thymic and pTregs in response to microbial components.

### Microbial regulation of thymic treg development

GF mice generally display a reduced thymus size, indicating the importance of microbiota in thymic cellular development and immune maturation.^5^ Within the thymus, medullary thymic epithelial cells (mTECs) play a central role in establishing central self-tolerance. They achieve this through the negative selection of self-reactive T cells via clonal deletion or their differentiation into Tregs. This process is facilitated by the promiscuous expression of tissue-restricted antigens (TRAs), driven by transcription factors such as Aire^46,47^ and Fezf2.^48^ Intriguingly, mTECs also express multiple Toll-like receptors (TLRs), suggesting a potential role for microbial signals in thymic Treg development.^49^ While TLR signaling has been shown to be important for Treg generation, there is no significant difference in TLR-MyD88-mediated cytokine gene expression between mTECs from GF and specific pathogen-free (SPF) mice. This indicates that mTEC TLRs may be activated by endogenous ligands rather than microbial signals.^49^

Both mTEC and thymic DCs can present antigens to drive Treg cell generation.^50,51^ During a critical period of early neonatal life in mice, intestinal CX3CR1^+^ dendritic cells transport microbial antigens from the intestine to the thymus. Interestingly, these antigens primarily stimulate microbiota-specific conventional T cells rather than tTregs.^52^ However, this study utilized Segmented filamentous bacteria (SFB) as a model microorganism, predominantly inducing Th17 T cell responses, thus it remains to be seen if Treg-inducing bacteria could expand tTregs under similar settings.

Further evidence of microbial influence on tTregs comes from studies using limited T cell receptor (TCR) models. The TCR repertoire of tTregs was found to be significantly overlapping with colonic Tregs,^37^ suggesting shared antigen specificity between these populations. In mice deficient in extra-thymic Treg generation, a niche of tTregs is established in early post-natal life. Interestingly, these cells proliferate independent of IL-2 signaling but require microbial antigens for their expansion, highlighting the role of microbial signals in shaping thymic Treg dynamics.^36^

Despite these insights, the precise contribution of microbial signals to thymic Treg development remains unclear. The lack of definitive markers and the interchangeability between Treg subsets make it challenging to unequivocally determine the thymic origin of microbiota-induced Tregs. Future studies employing lineage-tracing models and single-cell technologies, as well as monocolonization studies with Treg-inducing bacteria, will be essential to dissect the mechanisms by which microbial signals influence thymic Treg development and function.

### Microbial antigen-induced pTregs

Immune tolerance to gut microbiota is primarily mediated by peripheral RORγt^+^ Tregs (RORγt^+^ pTregs), which arise from naïve conventional CD4^+^ T cells under specific activation conditions.^6,7^ These RORγt^+^ pTregs populate the gut mucosal immune system during a critical developmental window around weaning in mice, coinciding with robust microbial colonization of the gut.^28^ The generation of these pTregs depends on bacterial antigens, diet-derived metabolites, and host-produced retinoic acid.^28^ Interestingly, disruptions to the microbiota during this early life period can lead to inflammatory pathologies later in life, underscoring the importance of this temporal window in establishing immune tolerance.^28^

Gut microbiota is essential for generating pTreg diversity and their functional fitness in the colon.^53^ Despite significant progress in understanding pTreg biology, the identity of the antigen-presenting cells (APCs) responsible for mediating their induction has remained elusive. CD103^+^ conventional dendritic cells (cDCs) have been implicated in promoting pTreg differentiation in response to luminal antigens.^20,54–59^ However, studies using adoptive transfer models have demonstrated that *Helicobacter*-specific T cells can differentiate into pTregs even in the absence of CD103^+^ DCs, suggesting that these cells are not indispensable for microbial antigen-driven pTreg generation.^60^

**Figure 1.**
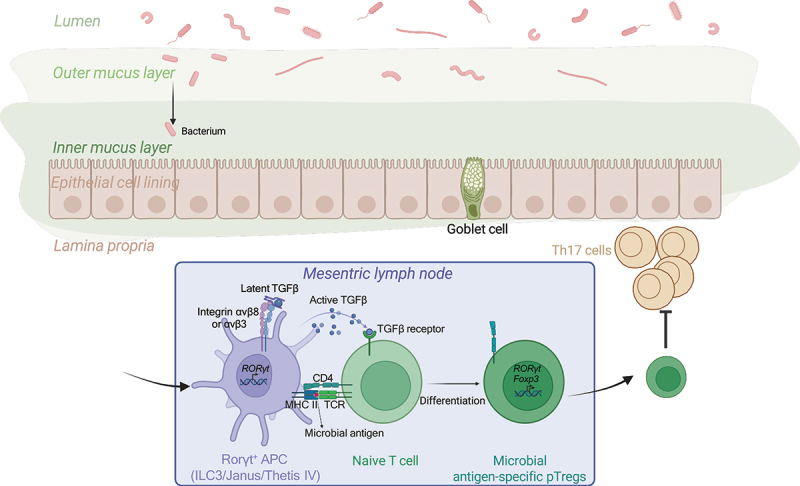
Mechanism of microbial antigen-specific pTreg generation in mesenteric lymph nodes.

Recent investigations have highlighted the potential role of RORγt-expressing APCs in pTreg induction^61–63^ (Figure 1). Consistent with previous observations,^60^ these studies excluded a role for conventional DCs in this process. Deletion of MHCII from RORγt^+^ APCs resulted in a marked reduction in gut RORγt^+^ pTregs. Lyu et al. identified lymphoid tissue inducer (LTi)-like group3 ILC (ILC3) as key players in RORγt^+^ pTreg generation through antigen presentation and integrin αvβ3-mediated processing of latent TGFβ.^63^ Notably, this study provided the first evidence implicating integrin αvβ3 in pTreg induction. In a mouse model where MHCII was deleted specifically in ILC3s (*H2-Ab1*^fl/fl^*Rorc*^Cre^), a significant reduction in RORγt^+^ pTregs was observed in mLNs and large intestine. Furthermore, a correlation between ILC3s and RORγt^+^ pTregs was observed in the human intestine, with a disruption of these cells noted in patients with IBD.^63^ Kedmi et al. demonstrated that RORγt^+^ APCs (which were either ILC3 or Janus type cells) require CCR7-mediated migration, MHCII-dependent antigen presentation, and integrin αvβ8 functionality to effectively induce RORγt^+^ pTregs.^62^ When these processes are impaired, the failure to generate pTregs results in the expansion of pathogenic Th17 cells instead.

In the gut, bacterial antigens are captured by RORγt^+^ antigen-presenting cells (APCs) that express either integrin αvβ8 or αvβ3, which activate latent TGF-β. These APCs present microbial antigens to naïve T cells in an MHC class II-restricted manner while simultaneously activating latent TGF-β. This process drives the differentiation of naïve CD4^+^ T cells into CD4^+^RORγt^+^Foxp3^+^ peripheral regulatory T cells (pTregs) in the presence of active TGF-β.

In contrast, Akagbosu et al. identified Thetis cells, a distinct subset of RORγt^+^ APCs, as key mediators of pTreg generation during early life. Specifically, subgroup TC IV, characterized by the expression of *Itgav* and *Itgb8* (encode Integrin subunit α_v_ and β_8_, respectively) and *Tgfb2* (encodes TGFβ), was shown to play an essential role in this process.^61^ In contrast to the findings reported by Lyu et al., Akagbosu et al. demonstrated that ILC3s were dispensable for RORγt^+^ pTreg generation using *H2-Ab1*^fl/fl^*Rora*^Cre^ mice, which selectively delete MHCII in ILC3. This discrepancy may arise from differences in Cre drivers used in the respective mouse models, suggesting the possibility that *Rorc*^Cre^-mediated deletion might also affect MHCII expression in Thetis cells. Resolving this issue will require the development of genetic tools to specifically target Thetis cells. Further, by analyzing the single-cell atlas of human intestinal and gut-draining lymph node cells spanning fetal to adult life,^64^ Akagbosu et al. identified a cluster within the myeloid cells that expressed signature Thetis cell genes – *TNFRSF11B* and *SPIB*, along with *AIRE*. These cells were predominantly localized in mLNs and enriched in fetal samples, suggesting a potential role in establishing gut immune tolerance early in life. Whether these cells functionally contribute to the establishment of tolerance to gut microbiota in humans remains to be determined. Future studies are needed to elucidate their mechanistic roles and validate their functional significance in peripheral immune tolerance.

The human leukocyte antigen (HLA) system plays a crucial role in shaping the interaction between microbial antigens and the host immune system, including the development of Treg cells. Certain HLA alleles have been associated with altered susceptibility to autoimmune and inflammatory conditions, which may be partly mediated through their influence on microbiota-Treg interactions.^65^ For instance, HLA-DQ2 and HLA-DQ8 haplotypes, which are strongly associated with celiac disease (CeD), influence the presentation of both gluten peptides and potentially microbial antigens that may share structural similarities.^66^ This molecular mimicry could affect Treg induction and function in genetically susceptible individuals. Furthermore, recent studies have demonstrated that specific HLA alleles can influence the composition of the gut microbiota,^67^ potentially creating a feedback loop that affects Treg homeostasis. The HLA-microbiota-Treg axis represents an important area for future research, particularly in understanding how genetic factors influence individual responses to microbial antigens and subsequent immune regulation.

### Microbe-derived ligands in treg generation

Bacterial structural components, such as lipopolysaccharides, peptidoglycans etc. interact with diverse host immune receptors, including TLRs and NOD-like receptors (NLRs), to shape the immune landscape. While the adjuvant effect of microbial components in activating the effector immune response is well established, we and others have demonstrated their equally critical role in driving immunoregulatory responses.^27,29,68,69^ Our previous work has demonstrated that a probiotic mixture named IRT5, comprising *Lactobacillus acidophilus*, *Lactobacillus casei*, *Lactobacillus reuteri*, *Bifidobacterium bifidum*, and *Streptococcus thermophilus*, induces the generation of Foxp3^+^ Tregs.^70^ This process is mediated by tolerogenic DCs that express high levels of IL-10, TGF-β, COX-2, and indoleamine 2,3-dioxygenase (IDO). Similarly, *Lactobacillus pentosus* KF340 (LP340) induced IL-10 Type 1 regulatory T cells (Tr1 cells), alleviating atopic dermatitis in mice.^71^ However, the specific effector components responsible for these immunomodulatory effects remained unidentified. Identifying these effector components is crucial for comprehending the molecular language of host-microbiome interactions. Moreover, this knowledge is essential for developing prebiotics, probiotics, and live biotherapeutic products (LBP) with a broad therapeutic window. To address this gap, we recently have rationally identified a unique dietary commensal strain, *Lactiplantibacillus plantarum* IMB19 (LpIMB19), and its effector component capsular rhamnose-rich heteropolysaccharide (RHP), which has the capability to enhance CD8 T cell immune response and augment anti-tumor immunity.^72,73^ The RHP functions as a TLR2 ligand, modulating tumor-associated macrophages toward an inflammatory phenotype, which subsequently activates CD8 T cells. To modulate Treg-mediated immunoregulatory responses, we and other researchers have identified specific microbial ligands capable of enhancing both the frequency and suppressive function of Tregs. These ligands have been shown to effectively alleviate disease progression in various mouse models of gut-related disorders as well as pathologies affecting distant tissues.

### Polysaccharide a (PSA)

**Figure 2.**
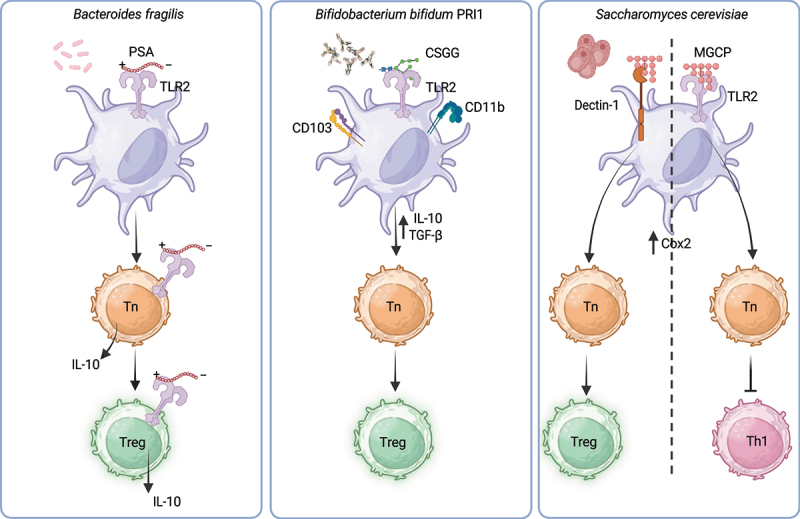
Microbial ligands drive pTreg generation and modulate immune responses.

In a significant study, Mazmanian et al.^28^ identified PSA, a protease-resistant zwitterionic capsular polysaccharide derived from the human commensal bacterium *Bacteroides fragilis*, as the first example of a unique symbiont molecule capable of promoting immunoregulatory responses. PSA was shown to directly interact with TLR2 on T cells, driving the induction and expansion of Tregs and, thus, suppressing the differentiation of pro-inflammatory Th17 cells^74^ (Figure 2). This discovery established a foundational framework for the rational identification of commensal bacteria with Treg-inducing properties, offering new avenues for modulating immune tolerance. However, subsequent studies revealed additional layers of complexity in PSA-mediated immunomodulation. In an in vitro co-culture system, Kreisman et al.^75^ demonstrated that human CD4^+^ T cells exposed to PSA in the presence of a mixed population of APCs differentiated into IL-10-producing Tr1 cells, which are distinct from Foxp3^+^ Tregs. Notably, Telesford et al.^76^ found that the ability of PSA to induce Foxp3^+^ Tregs was dependent on DCs, suggesting that DC-mediated processing and presentation of PSA are critical for its Treg-inducing effects. This finding underscores the critical role of DC-mediated processing and presentation of PSA in shaping its Treg-inducing effects and highlights how specific APC subsets influence the nature of the T cell response elicited by PSA. The clinical relevance of PSA-producing *B. fragilis* has been further emphasized by studies showing a reduced prevalence of actively PSA-producing strains in colonic biopsies from patients with IBD.^77,78^ These observations suggest that the loss of PSA-mediated immunoregulatory signals may contribute to the dysregulated immune responses characteristic of IBD, underscoring the therapeutic potential of PSA and PSA-producing bacteria in restoring immune homeostasis. Genomic screening has identified various commensal bacteria, including some pathogens, that produce capsular zwitterionic polysaccharides akin to PSA. Notably, *Bacteroides cellulosilyticus* DSM 14,838 was shown to protect against colitis in mice,^79^ underscoring zwitterionic polysaccharides as a promising class of immunomodulatory molecules for therapeutic use.

Left Panel: *Bacteroides fragilis* produces a protease-resistant zwitterionic capsular polysaccharide known as Polysaccharide A (PSA), which acts as a ligand for Toll-like receptor 2 (TLR2). Upon binding to TLR2, PSA induces dendritic cells (DCs) to adopt a regulatory phenotype, promoting the differentiation of naïve CD4+ T cells (Tn) into peripheral regulatory T cells (pTregs). Additionally, PSA can directly interact with TLR2 on both naïve T cells and Tregs, stimulating the production of the anti-inflammatory cytokine IL-10

Middle Panel: *Bifidobacterium bifidum* strain PRI1 (Bb PRI1) expresses cell surface β-glucan/galactan polysaccharides (CSGG), which are potent inducers of pTregs. CSGG binds to TLR2 on CD103^+^CD11b^+^ DCs, driving these cells toward a tolerogenic phenotype characterized by the production of IL-10 and TGF-β. This environment promotes the differentiation of naïve CD4^+^ T cells into CD4^+^Foxp3^+^ pTregs.

Right Panel: Polysaccharides derived from commensal yeast cell walls, such as mannan/β-1,6-glucan-containing polysaccharides (MGCP), are strong inducers of pTregs and inhibit the differentiation of inflammatory Th1 cells. MGCP operates through two distinct pathways in DCs:

1. Binding to Dectin-1 on DCs enhances Cox2 production, fostering a tolerogenic phenotype that supports the differentiation of naïve CD4^+^ T cells into CD4^+^Foxp3^+^ pTregs.

2. MGCP-treated DCs suppress Th1 cell differentiation and IFN-γ production in a Cox2-dependent manner. Notably, this suppression requires MGCP binding to TLR2 on DCs.

### Cell surface - β glucan/galactan polysaccharides (CSGG)

Through extensive ex vivo screening to identify bacteria capable of inducing pTregs, we discovered that *Bifidobacterium bifidum* strain PRI1 (*Bb* PRI1) possesses significant pTreg-inducing properties.^29^ *Bifidobacterium* species are well-documented for their ability to colonize the gut of breastfed infants early in life, playing a critical role in shaping the neonatal immune system.^80^ Notably, supplementation with *Bifidobacterium* has been shown to alleviate allergic inflammation in infants with dysbiotic gut microbiota compositions.^81^ In GF mice mono-colonized with Bb PRI1, the strain was found to promote the development of CD103^+^ CD11b^+^ regulatory DCs in the colon. Intriguingly, monocolonization of GF mice with Bb PRI1 induced colonic Tregs with relatively diverse TCR clonotypes. These pTregs were not only reactive to the bacterium itself but also expanded in response to dietary antigen OVA and bacterial flagellin. To further explore how Bb PRI1 influences the functional orientation of colonic Treg cells with distinct TCR repertoires, we conducted single-cell RNA sequencing and performed a comparative analysis of colonic Tregs from both SPF and GF mice.^53^ Our findings indicate that Bb PRI1 could alter the activation trajectory of colonic Tregs, promoting the emergence of a distinct phenotypic subset that is prevalent in SPF mice but absent in GF mice. Additionally, Bb PRI1 exposure facilitated the expansion of specific Treg clonotypes characterized by shared transcriptional features. The microbiota-driven colonic Treg subset, identified as PD-1− CXCR3^+^ Tregs, exhibited greater suppressive capacity than their counterparts from GF mice, demonstrated increased IL-10 production, and played a central role in modulating enteric inflammation in dextran sodium sulfate (DSS)-induced colitis.^53^

Fractionation of Bb PRI1’s cellular components revealed that its cell surface CSGG were critical mediators of Treg induction. CSGG is a complex mixture of neutral polysaccharides, including β-1–6-glucan, β-1–4-galactan, β-1–6-galactan, and β-galactofuranan, which collectively act as ligands for TLR2.^82^ Engagement of TLR2 by CSGG triggers DCs to produce IL-10 and TGF-β. CSGG acts as a ligand for TLR2, triggering DCs to produce the anti-inflammatory cytokines IL-10 and TGF-β, fostering an immunoregulatory environment (Figure 2). While CSGG’s ability to activate TLR2 and induce DC-mediated production of IL-10 and TGF-β has been established, further research is needed to elucidate the downstream signaling pathways activated by TLR2 engagement and their precise role in mediating these immunomodulatory effects. Importantly, CD4^+^ Foxp3^+^ Tregs induced by CSGG treatment demonstrated functional activity, effectively suppressing the progression of inflammatory colitis in mouse models. It is to be noted that in Tregs, TLR signaling can have context-dependent effects. TLR2 activation by certain bacterial lipopeptides can temporarily abrogate the suppressive function of Tregs by inducing a shift toward a Th17-like phenotype, characterized by reduced Foxp3 expression and increased IL-17 production.^83^ This effect is mediated through the MyD88-dependent activation of NF-κB and PI3K/Akt pathways, which inhibit Foxp3 function.^84^ Conversely, TLR2 signaling can also promote Treg expansion under certain conditions like CSGG treatment, highlighting the context-dependent nature of these pathways.^29,74^

### Mannan/β-1,6-glucan-containing polysaccharides (MGCP)

Commensal fungi constitute about 2% of human microbial biomass^85^ and play a key role in immune regulation.^86^ High-throughput sequencing techniques have revealed that the gut microbiome harbors over 50 genera of fungi, such as *Candida*, *Saccharomyces*, and *Cladosporium* species being among the most prevalent.^87^ Fungal dysbiosis is increasingly recognized as a key feature of IBD.^88–90^ Enhanced colonization of the intestine by *Candida* species and elevated production of anti-*Saccharomyces cerevisiae* antibodies, have been observed in patients with IBD.^91–93^ Interestingly, the immunomodulatory properties of beta-glucans appear to vary based on their chemical structure, exhibiting either pro-inflammatory or anti-inflammatory effects. Under steady-state conditions, polysaccharides containing β-1,3-glucan predominantly enhance pro-inflammatory responses.^94^ In contrast, a relatively less abundant class of cell surface polysaccharides, obtained from the fractionation of yeast cell wall components coupled with the enzymatic removal of β-1,3-glucan termed MGCP, has been shown to exert strong anti-inflammatory effects on the immune system.^27^ These MGCPs exhibit immunomodulatory properties by promoting the induction of Tregs while simultaneously suppressing the differentiation of IFN-γ-producing Th1 cells^27^ (Figure 2). Mechanistically, MGCP mediates Treg induction through the modulation of DCs in a Dectin-1-dependent manner and induces them to produce Cox2. Although Dectin-1 is traditionally associated with pro-inflammatory immune responses,^95^ our data suggest that it may function in a ligand-specific manner when interacting with MGCP, thereby promoting the generation of immunoregulatory Tregs. Intriguingly, the suppressive effect of MGCP on Th1 differentiation was found to be dependent on TLR2 signaling in DCs, as TLR2-deficient DCs failed to inhibit Th1 differentiation when co-cultured with MGCP and naïve CD4^+^ T cells. In vivo, MGCP demonstrated therapeutic potential by suppressing the progression of T-cell transfer colitis and experimental autoimmune encephalomyelitis (EAE), underscoring its ability to mitigate inflammatory and autoimmune conditions.

However, given that Treg potentiation can hinder anti-tumor immunity, we observed that MGCP treatment exacerbated tumor growth in a mouse melanoma model. These findings provide critical insights into the complex interplay between fungal-derived polysaccharides and the host immune system, with implications for both autoimmune diseases and cancer immunotherapy. Furthermore, they underscore the importance of characterizing multiple ligands derived from microbial structural components. Identification of MGCP reveals that seemingly opposing immunomodulatory ligands may coexist within the same microbe,^27^ potentially exerting their effects in a context-dependent manner to fine-tune immune responses.

## Bacterial metabolites in treg generation and function

### Short-chain fatty acids (SCFAs)

SCFAs are small organic molecules composed of fewer than six carbon atoms, primarily produced through the microbial fermentation of dietary fibers in the colon.^96,97^ The most prominent SCFAs – acetate (C2), propionate (C3), and butyrate (C4) – play essential roles in Treg development, expansion, and function.^98,99^ A comprehensive list of gut microbiota species associated with SCFA production is provided in Table 1.

**Table 1.** Bacterial strains involved with production of short-chain fatty acids.

S.No.	SCFA	Phylum	Species	Strain	References
1	Acetate	Verrucomicrobiota	*Akkermansia muciniphila*	ATCC BAA-835	Zhuge et al.^100^; Lakshmanan et al.^101^
2		Actinobacteriota	*Bifidobacterium longum*	JCM 1217	Fukuda et al.^102^; Yoon et al.^103^
3		Actinobacteriota	*Bifidobacteria adolescentis*	L2–32	O’Riordan et al.^104^; Rios-Covian et al.^105^
4		Firmicutes	*Blautia hydrogenotrophica*		Martin et al.^106^
5		Bacteroidetes	*Bacteriodes spp*		O’Riordan et al.^104^
6	Propionate	Bacteroidetes	*Bacteroides xylanisolvens*	GGCC_0124	
7		Verrucomicrobiota	*Akkermansia muciniphila*	ATCC BAA-835	
8		Verrucomicrobiota	*Akkermansia sp.*	GGCC_0220	
9		Bacteroidetes	*Bacteroides uniformis*	GGCC_0301	
10		Bacteroidetes	*Barnesiella sp.*	GGCC_0306	van der Lelie et al.^107^
11		Bacteroidetes	*Bacteroides massiliensis*	DSM 17,679	
12		Bacteroidetes	*Bacteroides stercoris*	DSM 19,555	
13		Bacteroidetes	*Barnesiella intestinihominis*	DSM 21,032	
14		Firmicutes	*Megamonas hypermegale*	DSM 1672	
15		Bacteroidetes	*Bacteroides thetaiotamicron*	VPI-5482/ATCC 29,148	O’Riordan et al.^104^; Wang et al.^108^
16		Firmicutes	*Faecalibacterium prausnitzii*	ATCC 27,766	Rios-Covian et al.^105^; Zhou et al.^109^
17		Bacteroidetes	*Bacteroides fragilis*		
18		Firmicutes	*Clostridium ramosum*		O’Riordan et al.^104^
19		Bacteroidetes	*Prevotella copri*		
20		Firmicutes	*Eubacterium rectale*	ATCC 33,656	Mukherjee et al.^110^
21		Firmicutes	*Megamonas funiformis*	DSM 19,343	
22	Butyrate	Bacteroidetes	*Bitterella massiliensis*	GGCC_0305	
23		Firmicutes	*Clostridium symbiosum*	GGCC_0272 ATCC 14,940	
24		Firmicutes	*Eubacterium callanderi*		
25		Firmicutes	*Intestinimonas butyriciproducens*	GGCC_0179	
26		Firmicutes	*Clostridium butyricum*	GGCC_0151	
27		Firmicutes	*Blautia producta*	DSM 2950	
28		Firmicutes	*Anaerostipes hadrus*	ATCC 29,173	
29		Firmicutes	*Anaerostipes caccae*	DSM 14,662	
30		Firmicutes	*Subdoligranulum variabile*	DSM 15,176	
31		Firmicutes	*Faecalibacterium prausnitzii*	DSM 17,677	van der Lelie et al.^107^; O’Riordan et al.^104^
32		Firmicutes	*Acidaminococcus intestini*	DSM 21,505	van der Lelie et al.^107^
33		Firmicutes	*Clostridium tyrobutyricum*		
34		Firmicutes	*Roseburia intestinalis*		O’Riordan et al.^104^
35		Firmicutes	*Roseburia inulinovorans*		
36		Firmicutes	*Eubacterium hallii*		
37		Firmicutes	*Eubacterium rectale*	ATCC 33,656	O’Riordan et al.^104^; Lu et al.^111^

SCFAs serve as key signaling molecules between gut microbiota and host immune cells. They act as ligands for G-Protein coupled receptors (GPCRs) – GPR41, GPR43, GPR109A, and Olfr78,^119–121^ and induce pTreg generation and proliferation. Studies on human GPRs reveal that propionate activates both GPR43 and GPR41, acetate predominantly targets GPR43, and butyrate exhibits selectivity for GPR41.^122^ Additionally, GPR109A, is specifically activated by butyrate and the vitamin niacin.^123^ GPR43 is coupled to both Gαi and Gαq proteins, activating phospholipase C, inhibiting adenylyl cyclase, and triggering intracellular calcium release.^122^ In Tregs, GPR43 signaling enhances mTOR activity and glycolysis, supporting cellular proliferation and functional fitness.^124^ GPR41, predominantly coupled to Gαi, inhibits cAMP production and activates ERK1/2 and p38 MAPK pathways.^125^ SCFA binding to GPR109A activates Gαi proteins to inhibit adenylyl cyclase and reduce cAMP levels.^126^ In dendritic cells, GPR109A signaling induces the expression of anti-inflammatory genes and promotes the production of retinoic acid and IL-10, creating a tolerogenic environment conducive to Treg differentiation.^127^

In the GF mice, oral supplementation with SCFAs, significantly increased the frequency of colonic Tregs.^99^ This effect was mediated through SCFA binding to GPR43, followed by inhibition of histone deacetylase (HDAC) activity,^99^ leading to enhanced acetylation at *Foxp3* gene locus. In an adoptive transfer model of T cell mediated colitis, *GPR43*^−/−^ CD4^+^ T cells failed to convert to Tregs upon treatment with SCFAs.^99^ However, conflicting evidence exists regarding the dependency on GPCRs for SCFA-mediated Treg modulation. For instance, Park et al. demonstrated that SCFA-induced Treg differentiation occurs independently of GPR41 or GPR43 but instead relies on direct HDAC inhibition.^128^ Notably, acetate, despite being a potent GPR43 agonist, failed to enhance pTreg differentiation. Further, butyrate can bind to GPR109A on colonic APCs and induce expression of *Il10* and *Aldh1a1* to induce differentiation of Tregs.

In the cell intrinsic manner, SCFAs can act as epigenetic regulator and were shown to inhibit HDAC activity and thus, enhance histone acetylation of *Foxp3* gene locus. This epigenetic modification enhances the accessibility of transcriptional machinery to promoter regions and conserved non-coding sequences (CNSs), such as CNS3, within the *Foxp3* locus. Chloroform-resistant microbial strains, including *Clostridium* species, were found to restore colonic Treg numbers in GF mice, an effect attributed to their robust production of butyrate. Indeed, dietary supplementation with butyrylated starch ameliorated CD4^+^ T cell-induced transfer colitis by enhancing colonic Treg generation. Butyrate enhanced histone H3 acetylation at both promoter and CNS3 of the *Foxp3* gene locus.^31^ Similar observations were reported by Arpaia et al., showing that oral butyrate potentiates colonic pTreg differentiation via SCFAs. Butyrate and propionate, but not acetate, increased histone acetylation on intronic CNS1 of *Foxp3* gene via HDAC inhibition.^30^

**Figure 3.**
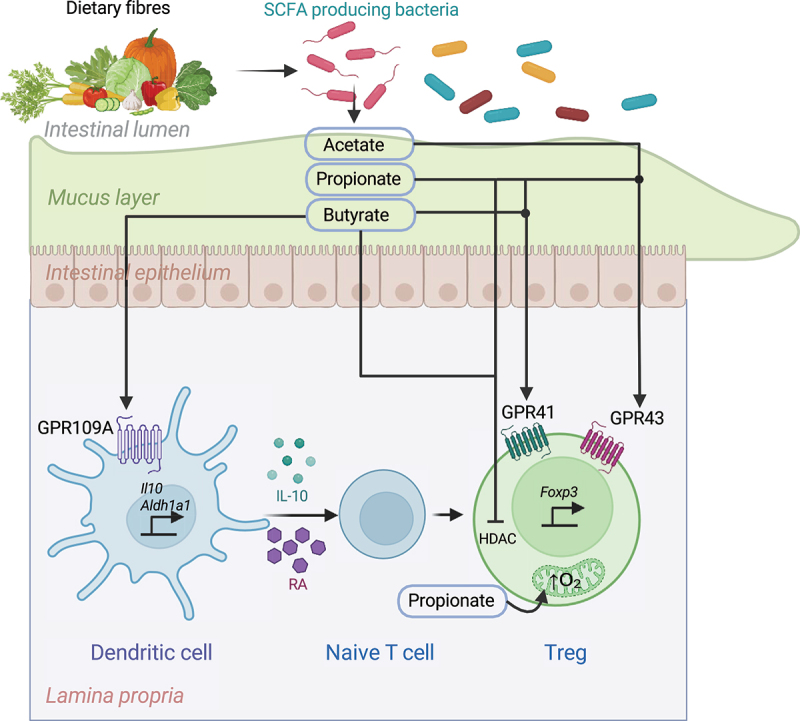
Mechanisms of intestinal treg modulation by microbial short-chain fatty acids (SCFAs).

Beyond their HDAC-inhibitory effects, SCFAs can promote Treg differentiation through metabolic reprogramming. For example, propionate treatment in patients with multiple sclerosis (MS) enhanced mitochondrial oxygen consumption rates, altered mitochondrial morphology, and boosted the suppressive functionality of Tregs. This treatment also increased the proportion of circulating Tregs, contributing to the mitigation of disease progression.^129^ These findings highlight the multifaceted mechanisms by which SCFAs modulate Treg biology, acting through both epigenetic and metabolic pathways (Figure 3).

Gut bacteria metabolize dietary fibers to produce short-chain fatty acids (SCFAs), including acetate, propionate, and butyrate, which play a critical role in the generation and function of intestinal peripheral regulatory T cells (pTregs).

Acetate: Acts as a ligand for the GPR43 receptor and inhibits histone deacetylase (HDAC) activity in Tregs upon binding to GPR43. This inhibition stabilizes Foxp3 expression, thereby enhancing the suppressive function of pTregs.

Propionate: Binds to both GPR41 and GPR43 receptors and similarly inhibits HDAC activity, contributing to the stabilization of Foxp3 expression. Propionate can also diffuse into Tregs to directly inhibit HDACs. Additionally, propionate enhances oxygen consumption in Treg mitochondria, improving their metabolic fitness and functional stability.

Butyrate: Functions as a ligand for GPR109A, expressed on gut dendritic cells (DCs). Activation of GPR109A upregulates the expression of *Il10* and *Aldh1a1* in DCs, leading to increased production of IL-10 and retinoic acid (RA). These factors promote the differentiation of naïve CD4^+^ T cells (Tn) into pTregs. Butyrate can also diffuse into Tregs to inhibit HDACs, further stabilizing Foxp3 expression.

In human studies, abnormal concentrations of SCFAs have been observed in various disease states, providing important clinical correlates to mechanistic findings in animal models. Patients with IBD consistently show reduced fecal SCFA levels, particularly butyrate, compared to healthy controls^130–132^ correlating with impaired Treg induction, increased mucosal inflammation, and disease exacerbation. This reduction correlates with decreased abundance of butyrate-producing bacteria such as *Faecalibacterium prausnitzii* and *Roseburia* species.^133^ Similarly, reduced SCFA levels have been reported in patients with multiple sclerosis,^134^ type 1 diabetes,^135^ and asthma,^136^ suggesting a common metabolic signature across multiple immune-mediated conditions. In mouse models, SCFA depletion in DSS-induced colitis exacerbates inflammation and reduces Treg populations, while butyrate supplementation ameliorates symptoms.^131^ Similarly, in murine CRC models, low butyrate promotes tumor growth, and in metabolic disease models, SCFA reductions impair insulin sensitivity.^6^ Interestingly, there are notable discrepancies between mouse models and human conditions regarding SCFA metabolism and effects. Owing to their fiber-rich diets and *Clostridia*-enriched microbiota, mice generate higher SCFA concentrations, while typical human diets produce substantially lower SCFA levels.^6,32,131,132^ Additionally, the distribution and expression patterns of SCFA receptors differ between mice and humans, potentially affecting downstream signaling pathways.^137^ For instance, GPR41 and GPR43 expression patterns in immune cells show species-specific differences, which may influence the immunomodulatory effects of SCFAs.^138^ These discrepancies highlight the importance of validating findings from mouse models in human studies and considering species-specific differences when translating basic research into clinical applications.

The impact of SCFAs on immune regulation appears to be highly context-dependent, with potentially divergent outcomes based on the local immune environment, concentration, and disease context.^139,140^ While SCFAs are widely recognized for their immunoregulatory functions by promoting Treg differentiation and function, their immunomodulatory effects can vary based on concentration, receptor engagement, and the local immune environment.^140^ Acetate has been shown to have limited effects on Treg differentiation compared to butyrate and propionate, and in some contexts, it may enhance pro-inflammatory responses by promoting effector T cell functions.^141–143^ High concentrations of butyrate can induce apoptosis in colonic epithelial cells, potentially compromising barrier integrity.^144,145^ Also, while promoting Treg differentiation in healthy contexts, butyrate can enhance oxidative stress and exacerbate inflammation in CRC by activating oncogenic Wnt/β-catenin signaling.^146^ Similarly, propionate amplifies Treg suppressive capacity in autoimmunity but may impair anti-tumor immunity by dampening CD8+ T cell responses.^147^

Furthermore, in certain neurological conditions, elevated SCFA levels have been associated with microglial activation and neuroinflammation, highlighting their dual nature.^148^ In EAE, studies have reported both protective and exacerbating effects of SCFA supplementation, suggesting complex regulatory mechanisms that may vary by disease stage and immunological context.^134,149^ These findings highlight the dose- and context-dependent duality of microbial metabolites, necessitating careful therapeutic targeting.

### Tryptophan metabolites

Tryptophan (Trp) is an essential aromatic amino acid for humans supplied by dietary proteins. The gut microbiome possesses diverse enzymes capable of processing dietary nutrients into a broad spectrum of metabolites, which could play an important role in host pathophysiology.^150^ Despite Trp being the least abundant amino acid in proteins and cells, it is a precursor to a wide variety of microbial and host metabolites.^151^ Dietary Trp is absorbed primarily in the small intestine and is metabolized through three major pathways. Approximately 90% of Trp is metabolized via Kynurenine pathway by host IDOs and tryptophan 2,3-dioxygenase (TDO) enzymes.^152,153^ This generates several biologically active metabolites like kynurenine (Kyn), kynurenic acid (Kna), 3-hydroxykynurenine (3-OHKyn), 3-hydroxyanthranilic acid (3HAA), and quinolinic acid.^154^ About 5% of Trp is used to synthesize serotonin by tryptophan hydroxylases (TPH1 and TPH2). Serotonin is further metabolized into melatonin through sequential enzymatic steps involving serotonin-N-acetyltransferase and acetylserotonin O-methyltransferase.^113^ Notably, 90–95% of serotonin resides in the gastrointestinal tract, predominantly within enterochromaffin cells.^112,155,156^ The remaining 5% of dietary tryptophan is catabolized by gut bacteria into indole and its derivatives, including indole-3-acetic acid (IAA), indole-3-propionic acid (IPA), and others via Indole pathway.^157^ This process is particularly prominent in the distal colon, as gradual depletion of carbohydrates from proximal to distal colon shifts bacterial metabolism toward protein fermentation.^158^ Additionally, certain bacterial species, such as *Lactobacilli*, can degrade Trp in the stomach and ileum of mice.^159^

### Serotonin

Serotonin (5-HT) has emerged as a critical mediator of immune regulation, particularly in the context of Tregs. Unlike T effector cells (Teffs), Tregs express key components of the serotonergic system, including the serotonin transporter (SERT), serotonin receptors (5-HT1a and 5-HT2), and enzyme tryptophan hydroxylase, which converts Trp into serotonin.^160^ While under microbial influence, the majority of serotonin is produced by enterochromaffin cells in the gut epithelium,^155^ certain bacterial species like *Streptococcus* spp., *Enterococcus* spp., *Escherichia* spp., *Lactobacillus plantarum*, *Klebsiella pneumonia*, and *Morganella morganii* – can also synthesize serotonin.^161–163^ A comprehensive list of bacterial species involved in serotonin biosynthesis is provided in Table 2.

**Table 2.** Gut bacterial strains involved in modulating gut serotonin.

S.No.	Phylum	Species	Strain	Role in Serotonin Production/Modulation	References
1	Firmicutes	*Clostridium* spp.		Stimulates enterochromaffin cells to produce serotonin via metabolites.	Yano et al.^112^
2	Firmicutes	*Lactobacillus plantarum*	Strain WCFS1	Modulates serotonin levels via tryptophan metabolism.	O’Mahony et al.^113^
3	Firmicutes	*Lactobacillus reuteri*	Strain ATCC PTA 6475	Influences serotonin levels through immune modulation.	O’Mahony et al.^113^
4	Actinobacteria	*Bifidobacterium infantis*	Strain 35,624	Modulates serotonin levels via immune modulation and tryptophan metabolism.	Desbonnet et al.^114^
5	Proteobacteria	*Escherichia coli*	Strain K-12	Produces serotonin directly by metabolizing tryptophan.	Wikoff et al.^115^
6	Firmicutes	*Enterococcus* spp.		Influences serotonin levels via metabolites affecting enterochromaffin cells.	Reigstad et al.^116^
7	Bacteroidetes	*Bacteroides* spp.		Produces short-chain fatty acids (SCFAs) that stimulate serotonin production in enterochromaffin cells.	Fukumoto et al.^117^
8	Firmicutes	*Streptococcus* spp.		May modulate serotonin levels through unclear mechanisms.	Lyte^118^

**Figure 4.**
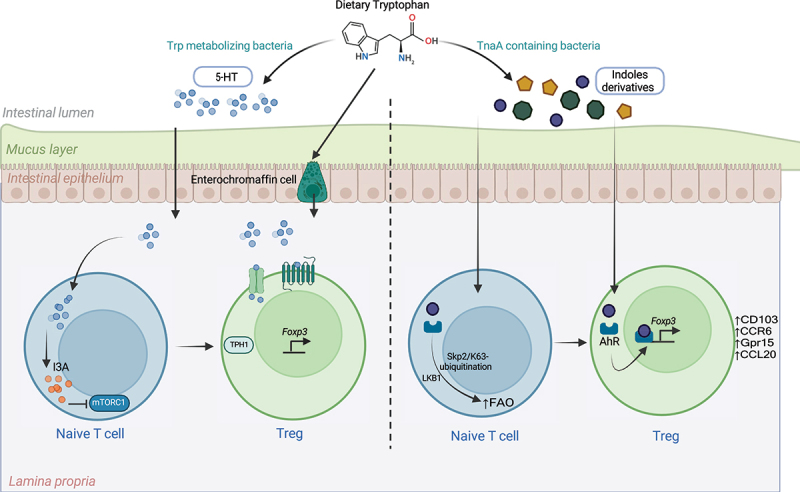
Mechanisms of intestinal treg modulation by microbial tryptophan metabolites.

In adult GF mice, serum and plasma levels of serotonin are significantly reduced, with the most pronounced deficits observed in the colon rather than the small intestine,^115,186^ suggesting a specific role of microbiota in regulating colonic 5-HT.^112^ However, recent studies have revealed that during early life, gut bacteria play a dominant role in serotonin production in the small intestine. For instance, *Rodentibacter heylii* and *Enterococcus gallinarum* contribute to serotonin synthesis in mice, while *Staphylococcus aureus*, *Clostridium perfringens*, *Klebsiella grimontii*, *Staphylococcus epidermis*, and *Enterobacter cloacae* perform similar functions in human small intestine.^187^ Mechanistically, this 5-HT inhibits mTORC1 in T cells via indole-3-acetaldehyde (I3A), promoting their differentiation into Tregs rather than effector T cells^187^ (Figure 4). Thus, bacterial serotonin facilitates the establishment of immune tolerance to dietary antigens and commensal microbes during early perinatal development. Oral administration of serotonin to neonatal mice followed by ovalbumin (OVA) sensitization induced long-term tolerance to OVA. Moreover, T cells from serotonin-treated mice exhibited enhanced tolerogenic properties in an adoptive transfer colitis model. Interestingly, serotonin treatment also altered gut microbiota composition, suggesting bidirectional regulation between the microbiome and Tregs via serotonin signaling.^187^

Dietary tryptophan is metabolized by gut bacteria through distinct pathways, generating bioactive compounds that modulate intestinal regulatory T cells (Tregs).

Serotonin Pathway: Bacteria containing tryptophan hydroxylase convert dietary tryptophan into serotonin (5-HT). Naïve CD4^+^ T cells take up serotonin and metabolize it into indole-3-acetaldehyde (I3A). I3A inhibits the mechanistic target of rapamycin complex 1 (mTORC1), promoting the differentiation of naïve T cells (Tn) into peripheral regulatory T cells (pTregs). Notably, Tregs themselves express serotonin receptors, transporters, and tryptophan-metabolizing enzymes, such as tryptophan hydroxylases (TPH1 and TPH2), suggesting a direct role for serotonin in Treg biology.

Indole Derivatives Pathway: Bacteria expressing tryptophanase (TnaA) catabolize tryptophan into various indole derivatives in the colon. These indole metabolites serve as ligands for the aryl hydrocarbon receptor (AhR). Activation of AhR enhance Liver kinase B1 mediated fatty acid oxidation via Skp2/K63-ubiquitination pathway in CD4^+^ T cells promoting Treg generation (left panel). Further, AhR activation enhances Foxp3 expression and gut homing molecules like CD103, CCR6, Gpr15, and CCL20 in peripheral Tregs. It reinforces the suppressive regulatory functions of Tregs, further promoting immune tolerance in the gut.

However, role of serotonin and Treg interaction in immune pathology remains complex and context-dependent. In arthritic mice deficient in serotonin, there is a shift toward Th17 cell polarization.^188^ Similarly, mice lacking enzyme Tph exhibit reduced Treg frequencies and increased Th17 responses during collagen-induced arthritis, effects that can be reversed by serotonin supplementation.^189^ However, in humans with allergic rhinitis, elevated serum serotonin levels correlate negatively with peripheral Treg frequencies, highlighting potential discrepancies between murine models and human disease states.^190^

### Indoles

Intestinal bacteria can convert the tryptophan into indole by enzyme tryptophanase (TnaA).^191^ Interestingly, in mammals, indole is produced exclusively through bacterial metabolism, as host cells lack the metabolic ability to synthesize it.^192^ While TnaA expression was earlier thought to be solely a characteristic of prokaryotes, recent evidence suggests that lateral gene transfer has enabled certain eukaryotic organisms, such as the gut-associated parasite *Blastocystis*, to acquire bacterial-derived TnaA,^193^ which could help its adaptation to gut environment.^194^

Beyond indole, the intestinal microbiota generates a diverse array of indole-related metabolites through tryptophan catabolism. These include indole-3-pyruvate, indole-3-lactate, indole-3-propionate, indole-3-acetate, indole-3-acetamide, indole-3-acrylate, indole acetaldehyde, indole-3-aldehyde, 3-methyl-indole (skatole), and indole-3-acetaldehyde.^195,196^ These metabolites play critical roles in maintaining intestinal barrier integrity, protecting against pathogens, and modulating host metabolism, primarily through the activation of the transcription factor aryl hydrocarbon receptor (AhR).^159,197,198^ A comprehensive list of bacterial species generating indole derivatives by Trp catabolism is provided in Table 3.

**Table 3.** Gut bacterial strains producing indole derivatives by tryptophan catabolism.

S.No.	Bacteria Phylum	Species	Indole Metabolite	References
1	Firmicutes	*Clostridium perfringens*	Indole	
2	Firmicutes	*Clostridium bifermentans*		
3	Firmicutes	*Clostridium tertium*		
4	Firmicutes	*Clostridium septicum*		
5	Firmicutes	*Clostridium histolyticum*		
6	Firmicutes	*Clostridium ramosum*		
7	Firmicutes	*Clostridium innocuum*		
8	Firmicutes	*Clostridium baratii*		
9	Firmicutes	*Clostridium paraputrificum*		
10	Firmicutes	*Clostridium beijerinckii*		
11	Firmicutes	*Clostridium acetobutylicum*		
12	Firmicutes	*Enterococcus faecalis*		
13	Firmicutes	*Clostridium sporogenes*		
14	Firmicutes	*Clostridium difficile*		
15	Firmicutes	*Clostridium butyricum*		
16	Bacteroidetes	*Bacteroides thetaiotaomicron*		Smith and Macfarlane^164^; Lee and Lee^165^; Buffie et al.^166^; Chen et al.^167^;Elsden et al.^168^; Devlin et al.^169^
17	Bacteroidetes	*Bacteroides ovatus*		
18	Firmicutes	*Clostridium limosum*		
19	Firmicutes	*Clostridium bifermentans*		
20	Firmicutes	*Clostridium malenomenatum*		
21	Firmicutes	*Clostridium lentoputrescens*		
22	Firmicutes	*Clostridium tetani*		
23	Firmicutes	*Clostridium tetanomorphum*		
24	Firmicutes	*Clostridium ghoni*		
25	Firmicutes	*Clostridium sordellii*		
26	Proteobacteria	*Desulfovibrio vulgaris*		
27	Firmicutes	*Enterococcus faecalis*		
28	Proteobacteria	*Escherichia coli*		
29	Fusobacteriota	*Fusobacterium nucleatum*		
30	Proteobacteria	*Haemophilus influenza*		
31	Firmicutes	*Peptostreptococcus asscharolyticus*		
32	Firmicutes	*Faecalibacterium prausnitzii*	Indole-3-propionic acid (IPA)	Sokol et al.^170^
33	Firmicutes	*Lactobacillus reuteri*	Indole-3-lactic acid (ILA)	
34	Firmicutes	*Lactobacillus plantarum*		
35	Firmicutes	*Lactobacillus casei*		
36	Firmicutes	*Lactobacillus acidophilus*		
37	Firmicutes	*Anaerostipes hadrus*		
38	Firmicutes	*Anaerostipes caccae*		
39	Bacteroidetes	*Bacteroides thetaiotaomicron*		
40	Bacteroidetes	*Bacteroides eggerthii*		
41	Bacteroidetes	*Bacteroides ovatus*		
42	Bacteroidetes	*Bacteroides fragilis*		
43	Actinobacteriota	*Bifidobacterium adolescentis*		
44	Actinobacteriota	*Bifidobacterium bifidum*		
45	Actinobacteriota	*Bifidobacterium longum* subsp. *infantis*		
46	Actinobacteriota	*Bifidobacterium longum* subsp. *longum*		
47	Actinobacteriota	*Bifidobacterium pseudolongum*		Zelante et al.^159^; Smith and Macfarlane^164^; Aragozzini et al.^171^; Cervantes-Barragan et al.^172^; Dodd et al.^173^; Honore et al.^174^; Russell et al.^175^; Wilck et al.^176^
48	Firmicutes	*Clostridium bartlettii*		
49	Firmicutes	*Clostridium perfringens*		
50	Firmicutes	*Clostridium sporogenes*		
51	Firmicutes	*Clostridium saccharolyticum*		
52	Proteobacteria	*Escherichia coli*		
53	Firmicutes	*Eubacterium rectale*		
54	Firmicutes	*Eubacterium cylindroides*		
55	Firmicutes	*Faecalibacterium prausnitzii*		
56	Firmicutes	*Lactobacillus murinus*		
57	Firmicutes	*Lactobacillus paracasei*		
58	Firmicutes	*Lactobacillus reuteri*		
59	Firmicutes	*Megamonas hypermegale*		
60	Bacteroidetes	*Parabacteroides distasonis*		
61	Firmicutes	*Peptostreptococcus asscharolyticus*		
62	Firmicutes	*Ruminococcus gnavus*	Indole-3-acetic acid (IAA)	
63	Firmicutes	*Roseburia spp.*		
64	Firmicutes	*Coprococcus comes*		
65	Firmicutes	*Blautia spp.*		
66	Firmicutes	*Clostridium scindens*		
67	Firmicutes	*Clostridium bartlettii*		
68	Firmicutes	*Clostridium hiranonis*		
69	Firmicutes	*Clostridium hylemonae*		
70	Firmicutes	*Clostridium sordellii*		
71	Bacteroidetes	*Bacteroides thetaiotaomicron*		
72	Bacteroidetes	*Bacteroides eggerthii*		
73	Bacteroidetes	*Bacteroides ovatus*		
74	Bacteroidetes	*Bacteroides fragilis*		
75	Actinobacteriota	*Bifidobacterium adolescentis*		
76	Actinobacteriota	*Bifidobacterium longum* subsp. *longum*		
77	Actinobacteriota	*Bifidobacterium pseudolongum*		Smith et al.,^164^ Elsden et al.,^168^ Russell et al.^175^; Li et al.^177^; Barbeyron et al.^178^; Valles-Colomer et al.,^179^; Zhu et al.^180^; Spanogiannopoulos et al.^181^
78	Firmicutes	*Clostridium bartlettii*		
79	Firmicutes	*Clostridium difficile*		
80	Firmicutes	*Clostridium lituseburense*		
81	Firmicutes	*Clostridium paraputrificum*		
82	Firmicutes	*Clostridium perfringens*		
83	Firmicutes	*Clostridium putrefaciens*		
84	Firmicutes	*Clostridium saccharolyticum*		
85	Firmicutes	*Clostridium sticklandii*		
86	Firmicutes	*Clostridium subterminale*		
87	Proteobacteria	*Escherichia coli*		
88	Firmicutes	*Eubacterium hallii*		
89	Firmicutes	*Eubacterium cylindroides*		
90	Bacteroidetes	*Parabacteroides distasonis*		
91	Firmicutes	*Peptostreptococcus asscharolyticus*		
92	Bacteroidetes	*Bacteroides thetaiotaomicron*	3-methylindole (Skatole)	
93	Firmicutes	*Butyrivibrio fibrisolvens*		
94	Firmicutes	*Clostridium bartlettii*		
95	Firmicutes	*Clostridium scatologenes*		
96	Firmicutes	*Clostridium drakei*		Russell et al.^175^; Honeyfield et al.^182^; Whitehead et al.^183^
97	Firmicutes	*Eubacterium cylindroides*		
98	Firmicutes	*Eubacterium rectale*		
99	Firmicutes	*Lactobacillus* spp.		
100	Firmicutes	*Megamonas hypermegale*		
101	Bacteroidetes	*Parabacteroides distasonis*		
102	Firmicutes	*Clostridium sporogenes*	Indoleacrylic acid (IA)	
103	Firmicutes	*Peptostreptococcus russellii*		Dodd et al.^173^; Wlodarska et al.^184^
104	Firmicutes	*Peptostreptococcus anaerobius*		
105	Firmicutes	*Peptostreptococcus stomatis*		
106	Firmicutes	*Lactobacillus acidophilus*	Indolealdehyde (IAld)	
107	Firmicutes	*Lactobacillus murinus*		Zelante et al.^159^; Cervantes-Barragan et al.^172^; Wilck et al.^176^
108	Firmicutes	*Lactobacillus reuteri*		
109	Firmicutes	*Clostridium botulinum*	Indolepropionic acid (IPA)	
110	Firmicutes	*Clostridium caloritolerans*		
111	Firmicutes	*Clostridium paraputrificum*		
112	Firmicutes	*Clostridium sporogenes*		
113	Firmicutes	*Clostridium cadvareris*		Wikoff et al.^115^; Elsden et al.^168^; Dodd et al.^173^; Wlodarska et al.^184^; Williams et al.^185^
114	Firmicutes	*Peptostreptococcus asscharolyticus*		
115	Firmicutes	*Peptostreptococcus russellii*		
116	Firmicutes	*Peptostreptococcus anaerobius*		
117	Firmicutes	*Peptostreptococcus stomatis*		
118	Firmicutes	*Clostridium sporogenes*	Tryptamine	Williams et al.^185^
119	Firmicutes	*Ruminococcus gnavus*		

AhR is expressed across multiple T cell subsets, with particularly high levels observed in Th17 cells, FOXP3^+^ Tregs, and Tr1 cells. Intriguingly, gut-resident Tregs exhibit elevated AhR expression compared to Tregs in other tissues, underscoring its specialized role in maintaining intestinal homeostasis and regulating gut Treg functions.^211^ Ahr activation affects Treg and Th17 development in a ligand-specific manner. For instance, 2,3,7,8-tetrachlorodibenzo-p-dioxin (TCDD), a xenobiotic AhR ligand, promotes Treg differentiation, while 6-formylindolo[3,2-b]carbazole (FICZ), an endogenous ligand derived from indole-3-acetaldehyde (I3AA) via bacterial metabolism, drives Th17 polarization.^212–214^ Thus, indole-mediated Treg differentiation and accumulation can be context-dependent and ligand-specific. Multiple AhR ligands can promote Treg development, leading to increased Treg numbers and improved outcomes in experimental autoimmune diseases.^215^ AhR activation enhances the expression of gut-homing molecules such as CD103, CCR6, Gpr15, and CCL20 in peripheral Tregs, facilitating their recruitment to the intestinal mucosa (Figure 4). Although AhR-deficient Tregs retain Foxp3 expression, they lose their suppressive functionality, emphasizing the critical role of AhR in Treg-mediated immune regulation.^216^ Interestingly, Ahr expression in intestinal Tregs is not dependent on microbiota, as GF or antibiotic-treated mice show no differences in Treg AhR levels.^216^

A phytochemical AhR ligand, indigo naturalis, has been shown to promote the accumulation of Helios^+^ Tregs near MHCII^+^ epithelial cells in intestinal crypts, further supporting the role of AhR in shaping the gut immune landscape.^217^ Additionally, AhR ligands enhance Liver kinase B1 mediated fatty acid oxidation via Skp2/K63-ubiquitination pathway in CD4^+^ T cells promoting Treg generation (Figure 4), which protect mice from DSS-induced colitis.^218^ In its inactive state, AhR resides in the cytoplasm as part of a complex with heat shock protein 90 (HSP90), AhR-interacting protein (AIP), and p23.^219^ Upon binding to ligands AhR undergoes conformational changes that expose its nuclear localization signal, leading to translocation into the nucleus.^213^ In the nucleus, AhR dimerizes with the AhR nuclear translocator (ARNT) and binds to specific DNA sequences known as xenobiotic response elements (XREs) in the promoter regions of target genes.^220^ In Tregs, AhR activation induces the expression of genes involved in Treg differentiation and function, including Foxp3, IL-10, and TGF-β.^221^ Additionally, AhR can interact with other transcription factors, such as c-Maf, to synergistically enhance IL-10 production.^222^ Furthermore, AhR activation in dendritic cells induces the expression of IDO1, creating a feedback loop that enhances kynurenine production and further activates AhR signaling.^223^

The interplay between microbial indole derivatives and Tregs remains an emerging area of research. A recent study demonstrated that the probiotic *Lactobacillus reuteri*, a producer of indole-3-lactate, cross-feeds other bacterial species and enhances microbial tryptophan metabolism.^224^ Elevated production of indole derivatives enriches the gut microbiota with *Clostridium* clusters XIVa, XIVb, and IV, known inducers of colonic Tregs.^225^ This microbial shift confers protection against *Citrobacter rodentium* infection and alleviates DSS-induced colitis.^224^

Conversely, disruptions in microbial indole metabolism can impair immune tolerance. Stephen-Victor et al. recently revealed that goblet-cell-derived resistin-like molecule β (RELMβ) influences the gut microbiome by depleting indole-metabolite-producing bacteria like *Lactobacilli* and *Alistipes*.^226^ This is achieved through the upregulation of antimicrobial genes such as *Sprr2a1/2/3* and *Reg3*, which alters the microbial balance, impairs oral tolerance, and exacerbates food allergy responses. *Lactobacilli* produce indole derivatives like IAA, I3A, and ILA, which promote the expansion of RORγt^+^ Tregs via AhR activation. In a mouse model of IL-4 receptor gain-of-function-induced food allergy, reintroducing *Lactobacilli* restored oral tolerance, whereas deleting AhR in Tregs abolished this protective effect.^226^ In conclusion, microbial indole derivatives and their interaction with AhR represent a critical axis in regulating intestinal immunity and Treg function. These metabolites not only shape the composition of the gut microbiota but also influence immune homeostasis and disease susceptibility. While significant progress has been made in elucidating the roles of indoles and AhR in immune regulation, further research is needed to fully unravel the intricate mechanisms underlying these interactions. Such insights hold immense therapeutic potential for modulating gut immunity and treating inflammatory and autoimmune disorders.

In humans, abnormal tryptophan metabolism has been observed in various inflammatory and autoimmune conditions. Patients with IBD show reduced serum levels of tryptophan and altered kynurenine pathway metabolites like indole-3-aldehyde, indicating enhanced IDO1 activity.^227^ Similarly, patients with multiple sclerosis exhibit altered tryptophan metabolism, with changes in the kynurenine-to-tryptophan ratio correlating with disease activity.^228^ Recent metabolomic studies have also identified reduced levels of AhR ligands in patients with psoriasis^229^ and rheumatoid arthritis,^230^ suggesting impaired tryptophan metabolism by the gut microbiota. Significant differences exist between mice and humans regarding tryptophan metabolism and AhR signaling. The affinity of various tryptophan metabolites for the AhR differs between species, with some ligands showing high potency in mice but limited activity in humans.^231^ Additionally, the expression patterns of enzymes involved in tryptophan metabolism vary between species, affecting the spectrum of metabolites produced.^159^ These differences may explain some of the challenges in translating AhR-targeted therapies from mouse models to human diseases. Future studies should focus on identifying human-specific AhR ligands and understanding their role in immune regulation to develop more effective therapeutic strategies.

### Bile acids

Bile acids (BAs) are amphipathic metabolites derived from cholesterol in the liver and play a crucial role in the digestion and absorption of dietary fats. Beyond their classical functions in lipid metabolism, BAs are now recognized as critical regulators of glucose and energy homeostasis.^232^ Further, identification of their receptors has paved the way for a deeper understanding of their hormone-like characteristics in regulating immune homeostasis.^233^

In humans, the liver synthesizes two primary BAs: cholic acid (CA) and chenodeoxycholic acid (CDCA). In contrast, rodents produce additional muricholic acids (MCA), which are 6-hydroxylated derivatives of CDCA.^234^ These primary BAs are conjugated with glycine or taurine in the liver before being secreted into the duodenum.^235,236^ Approximately 95% of secreted BAs are reabsorbed in terminal ileum and recycled back to liver via enterohepatic circulation. The remaining BAs enter the colon, where they undergo extensive microbial transformation.

Gut microbiota possessing bile salt hydrolase activity, such as bacteria from the genera *Lactobacillus*, *Bifidobacterium*, *Clostridium*, and *Bacteroides*, are able to deconjugate the BAs by cleaving the glycine or taurine moiety attached to the steroid core.^232,234^ Deconjugated BAs are further modified through dehydroxylation, epimerization, oxidation, desulfation, esterification, and reconjugation. For example, the dehydroxylation of CA and CDCA at the C7 position generates secondary BAs, including DCA and lithocholic acid (LCA), respectively. In mice, murideoxycholic acid is also formed from MCA.^237^ GF animals lack secondary BAs, underscoring the essential role of gut microbiota in bile acid metabolism.^238,239^ A list of gut bacterial strains involved in BA transformation reactions is provided in Table 4. BA-metabolizing enzymes help bacteria to overcome BA toxicity. Conversely, BAs help sustain microbial diversity, with human tauro-β-MCA and taurocholic acid playing key roles in shaping an adult-like microbiome.^240^ Dysregulation of bile acid metabolism, as seen in cholestasis or bile acid ligation models, is associated with reduced microbial diversity.^241,242^

**Table 4.** Gut bacterial strains involved in bile acid transformation reactions.

S.No.	Phylum	Species	Strain	Reference
1	Actinobacteria	*Bifidobacterium adolescentis*		Lucas et al.^199^
2	Actinobacteria	*Bifidobacterium bifidum*		
3	Actinobacteria	*Bifidobacterium dentium*		
4	Actinobacteria	*Collinsella aerofaciens*		
5	Actinobacteria	*Collinsella intestinalis*		
6	Actinobacteria	*Collinsella stercoris*		
7	Bacteroidetes	*Alistipes indistinctus*		
8	Bacteroidetes	*Bacteroides caccae*		
9	Bacteroidetes	*Bacteroides finegoldii*		
10	Bacteroidetes	*Bacteroides intestinalis*		
11	Bacteroidetes	*Bacteroides ovatus*		
12	Bacteroidetes	*Bacteroides thetaiotaomicron*	3731	
13	Bacteroidetes	*Bacteroides thetaiotaomicron*	7330	
14	Bacteroidetes	*Bacteroides thetaiotaomicron*	VPI-5482	
15	Bacteroidetes	*Bacteroides uniformis*		
16	Bacteroidetes	*Bacteroides vulgatus*		
17	Bacteroidetes	*Bacteroides xylanisolvens*		
18	Firmicutes	*Blautia hansenii*		
19	Firmicutes	*Blautia luti*		
20	Firmicutes	*Clostridium asparagiforme*		
21	Firmicutes	*Clostridium hylemonae*		
22	Firmicutes	*Clostridium leptum*		
23	Firmicutes	*Clostridium* M62_1		
24	Firmicutes	*Clostridium scindens*		
25	Firmicutes	*Coprococcus comes*		
26	Firmicutes	*Dorea formicigenerans*		
27	Firmicutes	*Dorea longicatena*		
28	Firmicutes	*Enterocloster bolteae* (formerly *Clostridium*)		
29	Firmicutes	*Erysipelatoclostridium ramosum* (formerly *Clostridium*)		
30	Firmicutes	*Holdemania filiformis*		
31	Firmicutes	*Hungatella hathewayi* (formerly *Clostridium*)		
32	Firmicutes	*Lactobacillus ruminis*		
33	Firmicutes	*Roseburia intestinalis*		
34	Firmicutes	*Ruminococcus* GM2/1		
35	Firmicutes	*Ruminococcus gnavus*		
36	Firmicutes	*Ruminococcus torques*		
37	Firmicutes	*Tyzzerella nexilis* (formerly *Clostridium nexile*)		
38	Fusobacterium	*Fusobacterium varium*		
39	Proteobacteria	*Escherichia coli*	K12 MG1655	
40	Proteobacteria	*Escherichia fergusonii*		
41	Proteobacteria	*Proteus penneri*		
42	Firmicutes	*Lactobacillus plantarum*	K21	Wu et al.^200^
43	Firmicutes	*Clostridium scindens*	ATCC 35,704	Ridlon et al.^201^; Wahlstrom et al.^202^
44	Actinomycetota	*Eggerthella lenta*		Doden et al.^203^
45	Firmicutes	*Ruminococcus gnavus*	ATCC 29,149	Doden et al.^203^
46	Firmicutes	*Bacillus subtilis*	R0179	Culpepper et al.^204^
47	Actinomycetota	*Bifidobacterium animalis subsp. lactis*	B94	Culpepper et al.^204^
48	Bacteroidetes	*Bacteroides fragilis*	NCTC 9343, ATCC 25,285	Sun et al.^205^
49	Firmicutes	*Lactobacillus salivarius*		Xu et al.^206^
50	Firmicutes	*Lactobacillus plantarum*	WCFS1, ATCC14197	Prete et al.^207^
51	Firmicutes	*Lactobacillus acidophilus*	ATCC 4356	Wu et al.^208^
52	Actinomycetota	*Eggerthella lenta*	DSM 2243, C592	Harris et al.^209^
53	Bacteroidetes	*Bacteroides thetaiotaomicron*	VPI-5482, ATCC 25,285	Adhikari et al.^210^
54	Bacillota	*Eubacterium rectale*	ATCC 33,656	Mukherjee et al.^110^

**Figure 5.**
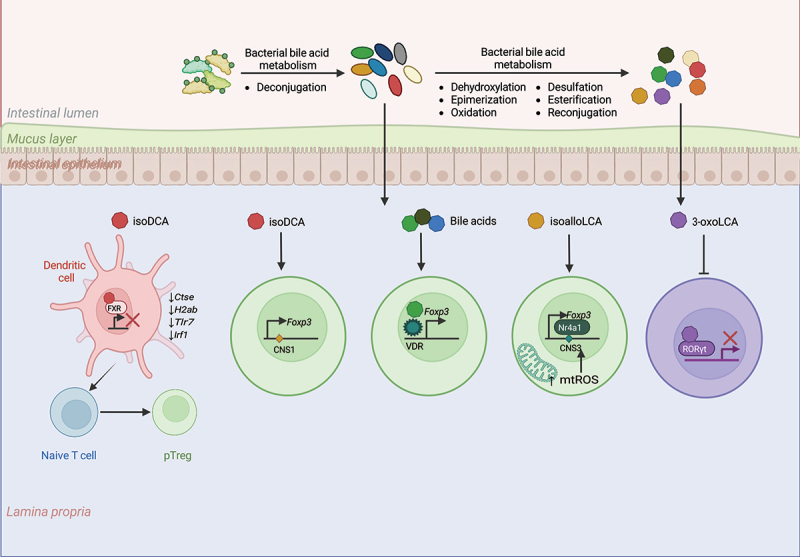
Mechanisms of intestinal treg modulation by microbial bile acid Metabolites.

BAs exert their immunomodulatory effects via a heterogenous family of transmembrane GPCRs and nuclear receptors. The nuclear receptor farnesoid X receptor (FXR) serves as the primary receptor for CDCA in humans and CA in mice,^243–245^ while secondary BAs like DCA and LCA activate G-protein bile acid receptor 1 (GBPAR1, also known as Takeda G-protein receptor (TGR5).^246^ Additionally, DCA and LCA interact with other nuclear receptors, including the vitamin D receptor (VDR),^247^ pregnane-X-receptor (PXR),^248^ and constitutive androstane receptor (CAR).^249^ Emerging evidence also implicates muscarinic M3 receptors^250^ and sphingosine-1-phosphate receptor^251^ in BA signaling (Figure 5).

Gut bacteria play a key role in bile acid (BA) metabolism by deconjugating primary bile acids that escape the enterohepatic circulation. These deconjugated BAs undergo further modifications by gut microbiota, generating secondary BAs that modulate intestinal Treg responses through diverse mechanisms.

isoDCA : This secondary bile acid metabolite inhibits the nuclear farnesoid X receptor (FXR) in dendritic cells (DCs), promoting their tolerogenic phenotype. These tolerogenic DCs facilitate the differentiation of naïve CD4^+^ T cells into CD4^+^RORγt^+^Foxp3^+^ pTregs(pTregs). Additionally, isoDCA enhances *Foxp3* expression in pTregs through a CNS1-mediated mechanism, further stabilizing their regulatory function.

Bile Acids and Vitamin D Receptor (VDR) : Certain bile acid metabolites act via the nuclear vitamin D receptor to upregulate *Foxp3* expression, driving the generation of pTregs and reinforcing immune tolerance.

isoalloLCA: A metabolite derived from lithocholic acid (LCA), isoalloLCA increases mitochondrial reactive oxygen species (ROS) in Tregs. This leads to the activation of the transcription factor Nr4a1 which enhances *Foxp3* expression through a CNS3-dependent mechanism, strengthening Treg suppressive activity.

3-oxoLCA: Another LCA-derived metabolite, 3-oxoLCA, suppresses Th17 cell differentiation by inhibiting RORγt binding, thereby reducing pro-inflammatory Th17 responses.

Several studies have highlighted critical role of BAs and their derivatives in regulating Treg differentiation in the intestine. Two derivatives of LCA − 3-oxoLCA and isoalloLCA – generated by bacterial modification of primary BAs have been shown to modulate T cell differentiation. 3-oxoLCA inhibits the differentiation of Th17 cells by directly binding to transcription factor RORγt. IsoalloLCA, on the other hand, increased mitochondrial reactive oxygen species (mtROS) leading to enhanced FOXP3 expression utilizing CNS3 enhancer region in the *Foxp3* locus.^32^ IsoalloLCA also promotes histone acetylation at the *Foxp3* promoter in the presence of TGF-β signaling (Figure 5).^32^ Subsequent work identified *Bacteroidetes* species as producers of isoalloLCA and demonstrated that its induction of mtROS generates Tregs via activation of nuclear receptor NR4A1 (Figure 5).^252^ Notably, patients with IBD exhibit reduced representation of genes encoding enzymes for isoalloLCA production in gut microbiome, along with decreased microbial synthesis of this metabolite.^252^ Human gut bacteria *Gordonibacter pamelaeae* P7-E3, *Eggerthella lenta* P7-G7, *Raoultibacter massiliensis* P7-A2, *Collinsella intestinalis* P8-C1, *Adlercreutzia equolifaciens* P11-C8 and *Clostridium citroniae* P2-B6 were later identified as top converters of LCA to 3-oxoLCA.^253^

BAs also expand pTregs through interactions with their receptors. Campbell et al. discovered that the secondary BA 3β-hydroxydeoxycholic acid (isoDCA) induces an anti-inflammatory phenotype in DCs by inhibiting the FXR activity, thereby promoting pTreg differentiation.^254^ The interaction between isoDCA and FXR downregulated several pro-inflammatory genes involved in antigen processing, presentation, and pro-inflammatory signal transduction in DCs (Figure 5). Furthermore, bacteria engineered to produce isoDCA enhanced colonic RORγt^+^ pTregs in a CNS1-dependent manner.^254^ Primary and secondary BAs can also induce RORγt^+^ pTregs by interacting with Treg-intrinsic VDR (Figure 5).^255^ This effect does not rely on Vitamin D3, as colonic RORγt^+^ pTregs were unaffected by its absence in diet but were significantly reduced by Treg-specific VDR deletion.

Overall, BAs, gut microbiota, and colonic pTregs form a dynamic and interdependent network essential for establishing intestinal immune tolerance. Intestinal BAs are indispensable for maintaining colonic pTregs, while gut microbes are instrumental in shaping this relationship by metabolizing BAs. Dysregulation of this triadic interaction can disrupt immune tolerance, contributing to inflammatory diseases such as IBD. Indeed, administration of BAs like LCA^255^ or rationally designed consortium composed of BA-producing bacteria^107^ have shown promise in reducing colitis severity.

Human studies have revealed significant alterations in bile acid profiles across various disease states. Patients with IBD show increased levels of primary bile acids and decreased secondary bile acids in feces, reflecting impaired microbial bile acid metabolism.^256^ This dysregulation is particularly pronounced in Crohn’s disease patients with ileal involvement, where bile acid malabsorption contributes to diarrhea and other symptoms.^257,258^ Similarly, patients with primary sclerosing cholangitis, which is often associated with IBD, exhibit distinct bile acid signatures characterized by elevated levels of toxic bile acids.^259^ Notable species differences exist in bile acid metabolism between mice and humans. Mice produce muricholic acids, which are potent FXR antagonists, whereas these bile acids are absent in humans.^260^ Additionally, the gut microbiota composition differs substantially between mice and humans, affecting the spectrum of secondary bile acids produced.^261^ These differences may explain some of the discrepancies observed when translating findings from mouse models to human conditions. For instance, while certain bile acid receptor agonists show promising results in mouse models of colitis, their efficacy in human IBD has been variable.^262^ Understanding these species-specific differences is crucial for developing targeted therapies based on bile acids for human diseases.

Additionally, microbial metabolites such as secondary BAs can have context-dependent effects, with some derivatives promoting inflammation under specific conditions.^263^ For instance, DCA has been implicated in pro-inflammatory responses in certain disease states, potentially exacerbating liver inflammation and colorectal cancer progression by inducing DNA damage.^264,265^ Furthermore, indole derivatives, while activating AhR-dependent Treg pathways, can also drive Th17 polarization in the presence of pro-inflammatory cytokines like IL-6.^197^ These findings highlight the dose- and context-dependent duality of microbial metabolites, necessitating careful therapeutic targeting.

## Impact of impaired immune responses on microbiota

While the influence of the microbiota on immune function has been extensively studied, the reciprocal impact of impaired immune responses on microbiota composition and function is equally important but less well characterized. Defects in Treg function or number can significantly reshape the intestinal microbial landscape, creating a dysbiotic environment that may further exacerbate immune dysregulation.^5^ Studies in mice with specific immune deficiencies have provided valuable insights into this relationship. For instance, mice lacking the anti-inflammatory cytokine IL-10, which is crucial for Treg function, develop spontaneous colitis accompanied by significant alterations in their gut microbiota, including increased abundance of pro-inflammatory Proteobacteria and decreased levels of beneficial Firmicutes.^266^ Similarly, Foxp3-deficient mice, which lack functional Tregs, exhibit profound dysbiosis characterized by the expansion of mucosa-associated segmented filamentous bacteria and other potentially pathogenic species.^267,268^

In humans, primary immunodeficiencies affecting Treg development or function, such as IPEX (Immune dysregulation, polyendocrinopathy, enteropathy, X-linked) syndrome caused by FOXP3 mutations, are associated with significant alterations in gut microbiota composition.^269^ These patients often exhibit reduced microbial diversity and increased abundance of opportunistic pathogens, which may contribute to their gastrointestinal symptoms and systemic inflammation. Beyond genetic immunodeficiencies, acquired impairments in immune function can also impact the microbiota. For example, HIV infection, which depletes CD4^+^ T cells including Tregs, leads to significant dysbiosis characterized by increased pathobiont abundance and reduced levels of beneficial bacteria.^270^ Similarly, immunosuppressive therapies used in transplantation and autoimmune diseases can alter the gut microbiota composition, potentially contributing to opportunistic infections and other complications.^271^

The mechanisms by which impaired immune responses affect the microbiota are multifaceted. Defects in antimicrobial peptide production, mucus layer integrity, and IgA secretion – all of which can be influenced by Treg function – directly impact microbial colonization and composition.^272^ Additionally, alterations in cytokine profiles and intestinal inflammation can create selective pressures that favor the expansion of certain bacterial species over others.^273^ This bidirectional relationship creates a potential feedback loop: impaired immune function leads to dysbiosis, which further exacerbates immune dysregulation, potentially contributing to chronic inflammation and disease pathogenesis. Understanding this complex interplay is crucial for developing targeted interventions that restore both immune homeostasis and a healthy microbiota.

## Dysregulation of microbiome-treg axis in diseases

### Inflammatory bowel disease (IBD)

#### Microbial dysbiosis and metabolite alterations in IBD

Dysbiosis, characterized by alterations in the diversity, composition, and function of the gut microbiota is a key aspect of IBD. The relationship between dysbiosis and IBD remains complex and bidirectional making it challenging to ascertain whether dysbiosis is a cause or consequence of the disease. Nonetheless, studies on GF mouse models have demonstrated that IBD either fails to develop or is significantly attenuated in the absence of gut microbes, underscoring the critical role of the microbiome in the pathogenesis of IBD.^274^ Genome-wide association studies have found that many of genomic loci associated with IBD are responsible for host-microbiome interactions.^275,276^ Gut bacterial -diversity significantly decreases in both ulcerative colitis (UC) and Crohn’s disease (CD) forms of IBD.^277,278^ Multi-omics^33^ and multi-biome^13^ analysis have revealed a consistent depletion of obligate anaerobes like *Faecalibacterium prausnitzii* and *Roseburia hominis*^13,33,279^ among other SCFA-producing bacteria like *Eubacterium* spp. (*E. rectale* and *E. ventriosum*), *Blautia* spp., *Bacteroides* spp., and *Anaerostipes hadrus*. Indeed, the IBD metabolome presented with a general reduction in SCFAs.^33^ Additionally, this was accompanied by a significant reduction in *Subdoligranulum* sp., which forms a complex of new species-level clade with at least seven butyrate producer species of *Subdoligranulum*, *Gemmiger*, and *Faecalibacterium* genera.^170,280^

An increase in primary bile acid cholate and its glycine and taurine conjugates was also observed in CD patients while secondary BAs lithocholate and deoxycholate were reduced.^33^ This shift suggests a depletion of secondary BA-producing bacteria or faster colonic transit times that limit microbial BA transformation in IBD patients.^256,281^ Additionally, an increase in fungal diversity has been reported in both UC and CD.^282^ IBD patients display an increased abundance of *Candida albicans* and a decreased abundance of *Saccharomyces cerevisiae*. However, *S. cerevisiae* was found enriched in a CD cohort in Japan and USA but was depleted in the China cohort.^13^ This suggests a geographical heterogeneity effect on IBD-associated mycobiome. Nevertheless, high levels of anti-*Saccharomyces cerevisiae* antibodies are robust biomarkers of CD.^278,283^

#### Treg dysfunction and therapeutic implications in IBD

Although IBD has a complex pathophysiology with the involvement of multiple factors, these findings indicate that dysbiosis-induced Treg dysfunction may play a role in IBD in genetically susceptible individuals, as both SCFAs and BAs are important for maintaining Treg-mediated gut immune tolerance. Indeed, colonization of GF mice with human fecal microbiota from IBD patients resulted in an increased number of Th17 cells and a reduced population of RORγt^+^ Tregs, compared to mice colonized with microbiota from healthy donors.^284^ Paradoxically, in human patients of CD colon lamina propria, Tregs are enriched while the circulating Tregs are decreased during active disease.^285–287^ Although Tregs present in the intestinal mucosa of IBD patients continue to express activation markers such as CTLA-4 and PD-1,^285,288^ these cells exhibit functional impairments and fail to effectively suppress inflammation.^286,289^ Notably, while Tregs derived from the mucosa of CD patients retain the ability to suppress peripheral CD4^+^ Teff cells isolated from blood, they are unable to exert similar suppressive effects on mucosal Teffs. Further, this finding suggests that gut-resident Teffs acquire resistance to Treg-mediated suppression during active IBD.^290^ Comprehensive single-cell analyses of intestinal tissues from various human IBD cohorts have uncovered distinct Treg subsets within the inflamed mucosa. These subsets exhibit a spectrum of Foxp3 expression and produce proinflammatory cytokines such as IL-17 and IFN-γ. Notably, a memory-like IL-17^+^ Treg population has been identified in patients with UC,^291^ alongside a TNF^+^ Treg subset,^292^ which might contribute to the anti-TNF treatment resistance in IBD patients.

Thus, schemes to expand functional mucosal Tregs or enhance their function can provide protection from IBD. Indeed, Treg expansion therapies like low-dose IL-2 treatment have been shown to provide moderate clinical response in UC patients with significant expansion of Tregs.^293^ Similarly, recent studies have demonstrated that microbial restoration through fecal microbiota transplantation (FMT) can improve outcomes in patients with UC form of IBD.^294–296^ Additionally, a defined consortium of probiotics, selected for their ability to produce beneficial metabolites such as SCFAs, indoles, and bile salts^107^ has demonstrated efficacy in ameliorating experimental colitis in murine models. This probiotic consortium not only reversed dysbiosis but also restored a functional gut microbiome capable of generating anti-inflammatory metabolites associated with mucosal homeostasis. Furthermore, it enhanced protective immunity by significantly increasing the frequency of IL-10-producing RORγt^+^ FoxP3^+^ Tregs. While microbe-derived products like PSA, CSGG, and MGCP have shown promising results in resolving experimental colitis in mice, clinical data remain limited. Nevertheless, given their ability to induce Tregs, it is reasonable to hypothesize that administrating these bioactive compounds from beneficial bacteria (postbiotics) could elicit favorable therapeutic responses in human IBD, warranting further investigation in clinical trials.

### Celiac disease (CeD)

#### Immune dysregulation and treg dysfunction in CeD

CeD is a chronic hyperimmune disorder caused by an abnormal immune response to gliadin, a component of gluten, in genetically predisposed individuals. Having compatible human leukocyte antigen (HLA) genetics is necessary for the development of CeD, but it alone does not cause the condition. While around 40% of the population possesses the permissive HLA genes, only approximately 3% of individuals develop CeD during their lifetime.^297^ This highlights the critical role of additional genetic, environmental, and immunological factors in disease pathogenesis. Though associated with changes in gut bacteria, a consistent microbial signature in patients has not been identified.^298^ The pathogenesis of CeD is known to primarily mediated by gluten-specific inflammatory Th1 and Th17 cells.^299,300^ Multiple studies have reported simultaneous expression of regulatory cytokines like IL-10 and TGF-β along with inflammatory cytokines IFN-γ, IL-17, and IL-21 in CeD.^301–303^ This creates a paradoxical environment in untreated CeD, where regulatory mechanisms attempt to suppress inflammation and mitigate the abnormal immune response triggered by gliadin.^304^

Studies have revealed intriguing parallels between CeD and IBD regarding Treg dynamics, as CeD is also characterized by an increase in Foxp3^+^ Tregs in small intestinal lamina propria.^305,306^ However, their suppressive functions are impaired significantly.^304,307,308^ IL-15 is significantly overexpressed in the intestines of celiac patients, where it contributes to immune dysfunction by disrupting TGF-β signaling, impairing Treg activity, and rendering Teff cells resistant to Treg-mediated suppression through activation of PI3K pathway.^309,310^ Additionally, Serena et al.^311^ highlighted the role of gut microbiome in the hypofunction of Tregs in CeD. In active CeD, the loss of intestinal barrier integrity allows microbial-derived butyrate to synergize with IFN-γ to modulate alternative splicing of *FOXP3*, favoring the expression of shorter *FOXP3* Delta 2 isoform, which lacks exon 2. This isoform compromises the interaction between FOXP3 and transcription factors RORαt and RORγt, thereby promoting Th17 differentiation.^312^ This shift in *FOXP3* isoform expression underscores how the intestinal microenvironment can reprogram Tregs, undermining their capacity to maintain immune tolerance and exacerbating the inflammatory response in CeD.

#### Microbiota alterations in CeD

The CeD-associated microbiota changes have been studied in high-risk infants with a first-degree relative diagnosed with CeD. These studies have revealed distinct microbial signatures, with increased abundance of the *Bacteroides-Prevotella* group,^313^ *Firmicutes*, *Proteobacteria*, and *Bifidobacterium* in infants compared to controls.^314^ Another study found that such infants exhibit a lower abundance of *Bacteroides* and a higher abundance of *Firmicutes* compared to healthy controls.^315^ In a longitudinal study, Olivares et al.^16^ observed that children who later developed CeD showed an increased abundance of *Firmicutes*, particularly *Enterococcaceae* and *Peptostreptococcaceae*, between 4 and 6 months of age. In contrast, no such differences were observed in control individuals during the same period. These findings suggest that early-life microbial dysbiosis may precede and potentially contribute to CeD pathogenesis.

A recent ongoing prospective clinical trial,^14^ utilizing shotgun metagenomic sequencing for functional characterization of microbes, Celiac Disease Genomic, Environmental, Microbiome and Metabolome study (CDGEMM), has further elucidated the relationship between environmental factors and microbial changes in high-risk infants. The study found that formula feeding was associated with an increased abundance of *Ruminococcus gnavus* and *Lachnospiraceae bacterium*, both of which have been linked to allergic and inflammatory conditions. Additionally, infants delivered by cesarean section exhibited a decreased abundance of *Bacteroides vulgatus* and *Bacteroides dorei*, alongside broader metabolomic alterations. One particularly intriguing finding from the CDGEMM study was the decreasing abundance of propionic acid in high-risk infants. Propionic acid is a known inducer of functionally competent Tregs.^129^ While it remains to be determined whether these microbial and metabolic changes directly contribute to CeD development, these findings underscore the potential importance of restoring Treg functionality or modulating the gut microbiome as novel therapeutic strategies.

Future research should focus on unraveling the precise mechanisms by which microbial and environmental factors influence immune regulation in CeD. Understanding these pathways could pave the way for innovative interventions aimed at restoring durable immune tolerance and preventing disease onset in genetically predisposed individuals.

### Colorectal cancer

#### Microbial alterations in CRC

Colorectal cancers (CRCs) are intrinsically linked to the gut microbiota due to their anatomical location within the gastrointestinal tract. Transplanting fecal microbiota from CRC patients into GF mice promotes colonic cell proliferation and accelerates colon tumor formation. Conversely, fecal microbiota from cancer-free individuals do not have the same effect, underscoring the role of CRC-associated microbiota in disease progression.^316^ A comprehensive multi-cohort metagenomic analysis identified a core bacterial signature of seven CRC-enriched bacterial species—*Bacteroides fragilis*, *Fusobacterium nucleatum*, *Porphyromonas asaccharolytica*, *Parvimonas micra*, *Prevotella intermedia*, *Alistipes finegoldii*, and *Thermanaerovibrio acidaminovorans*—that were consistently present across diverse populations spanning various geographies and ethnicities.^317^ In addition to these CRC-associated bacteria, the study also identified 62 bacterial species that were depleted in CRC patients. Notably, five of these depleted species—*Clostridium butyricum*, *Streptococcus salivarius*, *Streptococcus thermophilus*, *Carnobacterium maltaromaticum*, and *Lactobacillus gallinarum*—have been associated with health-promoting effects, underscoring their potential protective roles in the context of CRC development. Further, gut bacteria have been shown to modify response to immune check-point inhibitor therapy in multiple tumor types,^318–320^ including CRC.^321–323^ Fecal metagenomic and metabolomic data from individuals at various stages of colorectal tumorigenesis revealed dynamic changes in gut microbes and metabolites from early adenoma to the late stage of CRC suggesting dysbiotic changes could be drivers of CRC tumorigenesis.^324^

*F. nucleatum ssp. nucleatum*, *Solobacterium moorei*, *Peptostreptococcus stomatis*, *Peptostreptococcus anaerobius*, *Lactobacillus sanfranciscensis*, *Parvimonas micra*, and *Gemella morbillorum* are bacterial species that increased across all stages of tumor progression, while *Atopobium parvulum*, *Actinomyces odontolyticus*, *Desulfovibrio longreachensis*, and *Phascolarctobacterium succinatutens* were elevated only in early stages. Two butyrate-producing bacteria *Lachnospira multipara*, and *Eubacterium eligens* are significantly depleted in CRC. While relatively less studied, this loss of beneficial bacteria can be instrumental in CRC tumorigenesis. Furthermore, in the early stages of CRC, there is an increase in bile salt DCA, glycocholate, and taurocholate, indicating a role in tumorigenesis.^324^ Indeed, DCA increases DNA damage and mutations,^325^ while administration of BAs increases gut tumor incidences in mice.^326^

#### Tregs in CRC progression and therapy

Chronic inflammation is a well-established risk factor for the development and progression of various cancers, including CRC.^327^ The role of Tregs in this context presents a complex relationship with tumor progression. While they are pivotal in maintaining immune homeostasis and suppressing exuberant inflammation under normal conditions, their increased presence in tumors is implicated in cancer progression and indicates a worsening prognosis.^25,35^ Studies demonstrate that Tregs adopt a hyper-suppressive phenotype within TME, actively suppressing anti-tumor immunity and thus promoting CRC progression.^328,329^ These findings align with our recent demonstration that CRC-infiltrating Tregs exhibit enhanced activation of the NF-κB subunit C-REL, a Treg-effector transcription factor, driven by increased post-translational *O*-GlcNAcylation, which may contribute to their heightened immunosuppressive functions.^330^ However, some studies have reported that elevated densities of Foxp3^+^ Tregs correlate with suppression of CRC progression.^331,332^ These apparent contradictions may be explained by the heterogeneity of cells expressing FOXP3 in humans. Saito et al.^333^ identified a subset of FOXP3^lo^CD45RA-CD4^+^ TILs that transiently express FOXP3 but lack the canonical suppressive functions of bona fide Tregs. These cells are characterized by high expression of proinflammatory cytokines such as IL-17 and IFN-γ, suggesting that their accumulation in CRC may enhance anti-tumor immunity rather than suppress it and thus, their accumulation in CRC accentuates the anti-tumor immunity.^333^

Furthermore, considering the signature microbiota, which is depleted in the initiation stages of CRC being instrumental in colonic differentiation of RORγt^+^ Tregs and activation of colonic Tregs of thymic origin, it is highly probable that Tregs maintain a low inflammatory environment in the gut promoting intestinal immune homeostasis and thus, potentially inhibiting the tumorigenesis in the gut. Supporting this notion, a recent study by Frei et al.^334^ spatially resolved the immune markers over 3,000 CRC samples, distinguishing between intraepithelial and intrastromal compartments. Strikingly, they found that higher densities of intraepithelial CD8^+^ T cells and intrastromal Foxp3^+^ Tregs were strongly predictive of favorable clinical outcomes. The association of better prognosis with intrastromal rather than intraepithelial Tregs underscores their potential role in controlling inflammation and limiting tumor invasiveness. These findings suggest that enhancing the frequency and functionality of colonic Tregs through targeted interventions, such as specific probiotics, postbiotics, live biotherapeutic products, or microbial-derived ligands, could represent a promising therapeutic strategy for CRC. Characterizing the unique markers and mechanisms of stromal Tregs that inhibit tumor growth will be crucial for developing precise microbiome-based therapies. Such approaches could harness the immunoregulatory properties of Tregs to maintain gut immune homeostasis while simultaneously mitigating chronic inflammation, thereby offering a dual benefit in CRC prevention and treatment. Further research into the interplay between the gut microbiota, Treg biology, and tumor microenvironment dynamics will pave the way for innovative strategies aimed at modulating Treg activity to improve patient outcomes in CRC.

Despite the strong associations between microbiota alterations and various inflammatory and autoimmune diseases, establishing causality remains a significant challenge in the field. To distinguish whether dysbiosis is a cause or consequence of disease is inherently difficult due to the bidirectional nature of host-microbiome interactions.^335^ Studies in GF mouse models demonstrate that the absence of microbiota attenuates disease severity in IBD, suggesting a contributory role of the microbiome.^161^ Similarly, FMT from IBD patients to GF mice transfers disease phenotypes,^284^ however, reverse causality where inflammation itself reshapes the microbiota complicates interpretations. For example, intestinal inflammation reduces oxygen tolerance, favoring the expansion of facultative anaerobes like Proteobacteria.^336^ Moreover, clinical trials of probiotics and prebiotics have yielded mixed results *- Lactobacillus rhamnosus* GG ameliorates eczema but fails to prevent asthma,^337^ while high-fiber diets improve Treg responses in some IBD cohorts but show no benefit in others.^338^ Genetic polymorphisms in immune receptors (e.g., TLRs, NLRP3) further modulate individual responses to microbial signals, suggesting that microbiota-Treg interactions are heavily influenced by host factors.^276^ Moreover, geographical and genetic heterogeneity in microbial signatures, as observed with Saccharomyces cerevisiae in Crohn’s disease cohorts, underscores the challenge of establishing universal microbial drivers of disease.^13^ Furthermore, many studies reporting microbiome alterations in disease states are cross-sectional rather than longitudinal, limiting their ability to establish temporal relationships necessary for causal inference.^339^ These contradictory findings highlight the need for caution in interpreting the microbiota-Treg axis as uniformly beneficial. Future research leveraging longitudinal studies, multi-omics approaches, mechanistic studies, and controlled microbial interventions is essential to move beyond correlative observations and establish causal relationships in the microbiome-Treg axis and dissect the context-specific roles of microbial communities in immune regulation.

## Clinical translation: trials and challenges in targeting the microbiota-Treg axis

The promising results from preclinical studies targeting the microbiota-Treg axis have spurred numerous clinical trials, with varying degrees of success. Understanding both the successes and failures of these trials provides valuable insights for future therapeutic development.

Low-dose IL-2 therapy has emerged as a promising approach to expand Tregs in vivo. Several clinical trials have demonstrated that low-dose IL-2 can selectively expand Tregs without significantly affecting effector T cells in patients with various autoimmune conditions.^340^ In a phase 1/2 trial involving patients with ulcerative colitis, low-dose IL-2 treatment resulted in significant clinical improvement in 50% of patients, accompanied by expansion of FOXP3^+^ Tregs.^341^ However, challenges remain regarding the optimal dosing regimen, potential off-target effects, and long-term efficacy of this approach. Similarly, adoptive Treg transfer represents another strategy to restore immune tolerance. Early-phase clinical trials have demonstrated the safety and feasibility of ex vivo expanded autologous Tregs in conditions such as type 1 diabetes^342^ and Crohn’s disease.^343^ However, a phase 1 trial of ovalbumin-specific Tregs in Crohn’s disease patients failed to show significant clinical benefit despite demonstrating safety.^341^

FMT has shown promise in recurrent *Clostridioides difficile* infection and is being investigated for various immune-mediated conditions. In ulcerative colitis, several randomized controlled trials have demonstrated modest efficacy of FMT in inducing clinical remission.^344,345^ A trial of FMT in Crohn’s disease showed moderate benefit,^346^ highlighting the disease-specific effects of this approach. The variability in donor stool composition, optimal administration protocols, and long-term safety concerns remain significant challenges for FMT.

Probiotic interventions have yielded mixed results in clinical trials. While some studies have shown modest benefits of specific probiotic strains in conditions such as ulcerative colitis,^347,348^ others have failed to demonstrate significant effects as in atopic dermatitis.^349^ A notable failure was the PROPATRIA trial, which found that a probiotic mixture increased mortality in patients with severe acute pancreatitis,^350^ highlighting the potential risks of untargeted microbial interventions in certain clinical contexts. Postbiotic interventions, using microbial-derived components or metabolites, represent an emerging approach with potential advantages over live bacterial therapies. Early-phase trials of SCFA supplementation^351^ has shown promising effects on immune parameters, but larger efficacy trials are still needed.

However, clinical trials specifically examining the relationship between Tregs and microbial interventions remain limited, with most evidence coming from preclinical models or observational studies. Though several studies are investigating FMT in immune-mediated conditions, only few directly measured Treg outcomes. Al et al.^352^ conducted a pilot randomized controlled trial of FMT in multiple sclerosis patients (NCT03183869), measuring peripheral blood cytokines as the primary outcome. While this trial demonstrated that FMT was safe and tolerable, with potential to improve intestinal permeability and enrich for an MS-protective microbiota, it did not specifically report Treg changes. Similarly, NCT02516384 examined two donor FMT in ulcerative colitis patients with immunological assessments.^353^ Interestingly, along with moderate improvement in clinical response they found that both mucosal Th1 cells and Tregs were decreased post-FMT. Reduction in Tregs probably happened concomitant to reduction in mucosal inflammation as a result of increased microbial diversity. Preclinical evidence suggests that microbial interventions can influence Treg populations, as demonstrated in murine models where defined microbiota transplants restored Th17/RORγt^+^ regulatory T cell balance, but human clinical trial data with direct Treg outcome measurements remains an important gap in the current literature.

Furthermore, oral consumption as a substitute for bacterial functionality presents both opportunities and challenges. While oral administration of bacterial metabolites like SCFAs, tryptophan derivatives, or BAs could theoretically bypass the need for a functional microbiota, several limitations exist. These include the poor stability of many metabolites in the gastrointestinal tract, challenges in achieving physiologically relevant concentrations at target sites, and the loss of context-dependent production of these metabolites.^354^ Additionally, many bacterial functions involve complex metabolic networks and cell-to-cell interactions that cannot be easily replicated by single metabolites.^355^ Despite these challenges, targeted delivery systems and synthetic biology approaches are being developed to overcome some of these limitations. For example, engineered bacteria designed to produce specific metabolites or immune-modulating molecules in response to environmental cues represent a promising approach to combine the advantages of live bacteria with the specificity of postbiotic interventions.^356^

The mixed results from clinical trials targeting the microbiota-Treg axis highlight the complexity of translating preclinical findings to human diseases. Future success will likely depend on more personalized approaches that consider individual variations in microbiota composition, genetic factors, and disease heterogeneity. Additionally, combination therapies that target multiple aspects of the microbiota-Treg axis may prove more effective than single interventions.

## Conclusion and future perspectives

The interplay between the gut microbiome and Tregs represents a cornerstone of immune homeostasis, with profound implications for health and disease. This review has highlighted the multifaceted mechanisms by which microbial components and metabolites shape Treg development, differentiation, and function. These microbial-derived signals not only maintain intestinal immune tolerance but also influence systemic immunity, underscoring the gut microbiome’s role as a key modulator of immune responses.

Dysregulation of the microbiome-Treg axis is a hallmark of inflammatory and autoimmune diseases. In IBD, microbial dysbiosis and reduced production of immunomodulatory metabolites, such as SCFAs, impair Treg function, leading to chronic inflammation.^33^ Similarly, emerging evidence suggests microbiome-based changes in other conditions, such as MS and autism spectrum disorders (ASD), among others. In MS, alterations in gut microbial composition have been linked to immune dysregulation and disease progression,^357,358^ while in ASD, gut microbiome imbalances correlate with behavioral and neurological symptoms.^359^ In cancer therapy, specific microbial signatures have been identified as predictors of response to immune checkpoint inhibitors, highlighting the potential for microbiome modulation to enhance treatment efficacy.^360,361^ However, in many instances, it remains unclear whether microbial changes are a cause or consequence of disease processes, necessitating further investigation to establish causal relationships and mechanistic insights.

Emerging evidence suggests that targeting the microbiome-Treg axis holds immense therapeutic potential. Strategies such as FMT, probiotics, postbiotics, LBPs, and microbial-derived ligands have shown promise in preclinical and clinical studies.^107,294^ However, translating these findings into effective therapies requires a deeper understanding of the complex interactions between microbial signals, host immunity, and disease-specific contexts.

From a therapeutic perspective, FMT has emerged as a well-established approach for modulating the gut microbiota and correcting dysbiosis. Indeed, FMT-related therapeutics have been approved by the US FDA for recurrent *Clostridioides difficile* infections.^362,363^ However, defined consortia of bacteria offer significant advantages over FMT. These consortia can mimic the natural complexity of the gut microbiome, provide functional redundancy to ensure therapeutic stability, and promote stable colonization, potentially leading to long-term effects.^364^ Moreover, they can simultaneously target multiple pathways, making them suitable for complex diseases. However, the use of live biotherapeutics presents challenges, including variable responses in heterogeneous patient populations and inconsistent efficacy outcomes, necessitating rigorous investigation and well-designed clinical trials to address these limitations.^365^ Both LBP and FMT efficacy is highly context-dependent, influenced by factors such as donor and recipient microbiota composition, host immune status, host genetics, and delivery methods, which may limit long-term benefits.^366,367^ For instance, FMT trials in ulcerative colitis show variable remission rates due to differences in donor microbial profiles and patient baseline microbiota which may either facilitate or inhibit colonization by the introduced strains.^368,369^ Similarly, LBP outcomes, such as those with VE303, vary based on colonization success and host factors.^370^ The complex ecological dynamics within the gut microbiota, including competition for nutrients and niches, cross-feeding relationships, and antagonistic interactions, further complicate the predictability of microbiota-based interventions.^371^ These findings underscore the need for personalized approaches and further research to optimize donor selection, delivery protocols, and patient stratification to achieve sustained therapeutic outcomes.

In contrast, purified microbial products, such as PSA, CSGG, MGCP, RHP,^27,29,73^ and other microbial-derived ligands, may offer a more controlled and precise approach. These well-defined products enable consistent outcomes and facilitate the study of precise mechanisms, providing better control over therapeutic interventions. Logistically, purified products, if they have a simple chemical structure, might be safer, easier to manufacture and store, and face fewer regulatory hurdles compared to live consortia or FMT. Despite these advantages, the exploration of microbial products is still in its infancy, and a plethora of bioactive molecules remain to be discovered for various dysbiotic diseases. Additionally, the roles of understudied components of the human microbiome other than bacteria, such as fungi and viruses, in Treg regulation warrant further investigation, as they may hold untapped therapeutic and biomarker potential.^372–374^

Future research should focus on elucidating the precise molecular mechanisms by which microbial components and metabolites modulate Treg biology. Personalized microbiome-based therapies, tailored to individual microbial and immune profiles, could improve treatment outcomes and pave the way for precision medicine in immune-mediated diseases.^375^ Furthermore, the integration of multi-omics approaches, including metagenomics, metabolomics, and single-cell sequencing, will provide deeper insights into the microbiome-Treg axis and its role in health and disease.^376,377^

In conclusion, the microbiome-Treg axis represents a dynamic and bidirectional relationship that is central to immune homeostasis and disease. The context-dependent nature of microbial effects on immune regulation necessitates personalized approaches that consider individual variations in microbiota composition, host genetics, and disease pathophysiology.^378^ Moreover, the complex interplay between beneficial and potentially harmful microbial signals requires careful consideration when developing microbiota-based therapeutics. As demonstrated by failed clinical trials with FMT in ulcerative colitis, not all patients respond uniformly to microbiome-targeted interventions, highlighting the need for better stratification approaches and more precise manipulation of specific microbial pathways.^345^ Future research should focus on establishing causality through longitudinal studies, identifying disease-specific microbial signatures with strain-level characterization, metabolite profiling in disease-specific contexts, integration of multi-omics data, and developing targeted approaches to modulate specific aspects of the microbiome-Treg axis while minimizing unintended consequences. By unraveling the complexities of this interaction, we can harness the therapeutic potential of the microbiome to restore immune tolerance and improve outcomes in inflammatory, autoimmune, and neoplastic diseases. The development of microbiome-based therapies, whether through live consortia, purified products, or personalized interventions, holds immense promise for revolutionizing the treatment of immune-mediated disorders.
